# Disease candidate gene identification and prioritization using protein interaction networks

**DOI:** 10.1186/1471-2105-10-73

**Published:** 2009-02-27

**Authors:** Jing Chen, Bruce J Aronow, Anil G Jegga

**Affiliations:** 1 Division of Biomedical Informatics, Cincinnati Children's Hospital Medical Center, Cincinnati, OH, USA; 2 Department of Biomedical Engineering, University of Cincinnati, Cincinnati, OH, USA; 3 Department of Pediatrics, University of Cincinnati College of Medicine, Cincinnati, OH, USA

## Abstract

**Background:**

Although most of the current disease candidate gene identification and prioritization methods depend on functional annotations, the coverage of the gene functional annotations is a limiting factor. In the current study, we describe a candidate gene prioritization method that is entirely based on protein-protein interaction network (PPIN) analyses.

**Results:**

For the first time, extended versions of the PageRank and HITS algorithms, and the K-Step Markov method are applied to prioritize disease candidate genes in a training-test schema. Using a list of known disease-related genes from our earlier study as a training set ("seeds"), and the rest of the known genes as a test list, we perform large-scale cross validation to rank the candidate genes and also evaluate and compare the performance of our approach. Under appropriate settings – for example, a back probability of 0.3 for PageRank with Priors and HITS with Priors, and step size 6 for K-Step Markov method – the three methods achieved a comparable AUC value, suggesting a similar performance.

**Conclusion:**

Even though network-based methods are generally not as effective as integrated functional annotation-based methods for disease candidate gene prioritization, in a one-to-one comparison, PPIN-based candidate gene prioritization performs better than all other gene features or annotations. Additionally, we demonstrate that methods used for studying both social and Web networks can be successfully used for disease candidate gene prioritization.

## Background

Most of the current disease candidate gene prioritization methods [1-6] rely on functional annotations. However, the coverage of the gene functional annotations is a limiting factor. Although more than 1,500 human disease genes have been documented, most of them continue to be functionally uncharacterized. Currently, only a fraction of the genome is annotated with pathways and phenotypes [6]. While two thirds of all the genes are annotated by at least one functional annotation, the remaining one third is yet to be annotated.

Analysis of protein-protein interaction networks (PPINs) is important for inferring the function of uncharacterized proteins. Protein-protein interactions refer to the association among the protein molecules and the study of these associations from the perspective of biochemistry, signal transduction and biomolecular networks. Recent biotechnological advances like the high-throughput yeast two-hybrid screen facilitated building proteome-wide PPINs or "interactome" maps in humans [7,8]. The shift in focus to systems biology in the post-genomic era has generated further interest in PPINs and biological pathways. Network-based analyses have been developed with a number of goals [9], including protein function prediction [10], identification of functional modules [11], interaction prediction [12,13], identification of disease candidate genes [14,15] and drug targets [16,17], and the study of network structure and evolution [18-22]. While there is a wealth of protein-disease relationships in the published literature and a number of PPIN resources, relatively few studies have actually used PPIN analyses for prioritizing disease genes. Thus, making use of these networks in the context of disease is a relatively new challenge [14]. One of the earliest efforts [23] uses a classifier based on several topological features, including degree (the number of links to the protein), 1N index (proportion of links to disease-related proteins), 2N index (average 1N index in the neighbors), average distance to disease genes, and positive topology coefficient (average neighborhood overlapping with disease genes). Xu et al., built a KNN-based classifier with all disease genes from OMIM and concluded that hereditary disease genes from OMIM in the literature-curated protein-protein interaction network are characterized by a larger degree, a tendency to interact with other disease genes, more common neighbors, and quick communication to each other [23]. A more recent application, Genes2Networks [24], identifies important genes based on a list of "seed" genes. It generates a Z-score for each "intermediate" gene from a binomial proportions test to represent its specificity or significance to the "seed" genes. The former method, independent of known disease-related genes, is used for disease candidate gene identification, especially in cases where there is little or no prior knowledge about the disease. The latter application, on the other hand, uses a "seed" list as training to score the neighboring genes. It avoids bias toward highly connected "hub" genes, but the candidate gene is searched in a local network region, and the user has to provide the size of the neighborhood region in the network.

Recent technological advances in genomic sequencing, gene expression analysis, and other massively parallel techniques, while opening new opportunities, continue to pose a formidable challenge in deriving meaningful information from the large data silos. Typically, such data can be represented as networks in which the nodes (e.g., genes, mRNA, microRNA, proteins or metabolites) are linked by edges (e.g., DNA-protein or protein-protein or miRNA-mRNA interactions or correlations). Structural analysis of these networks can lead to new insights into biological systems and is a helpful method for proposing new and testable hypotheses. Biological networks have in fact been found to be comparable to communication and social networks [25]. For instance, PPINs and communication networks share several common characters such as scale-freeness and small-world properties, suggesting that the algorithms used for social and Web networks are equally applicable to biological networks. Although PPIs have been used widely to identify novel disease candidate genes [14,15,23,26-35], besides Kohler et al. [30] and Wu et al. [34], there have been no reports on using PPIs for disease gene prioritization. Additionally, to the best of our knowledge, this is the first study that uses social- and Web- network analysis-based algorithms to prioritize disease candidate genes.

In the analysis of social networks, Web graphs and telecommunication networks, a common question frequently asked is: Which entities are most important in the network? Although visualization-centered approaches such as graph drawing are useful to gain qualitative intuition about the structure, especially in small graphs, it is not practical to use these approaches for large and more complex networks. As an alternative, a number of other approaches have therefore been developed. For instance, a variety of measures (degree centrality [36], closeness centrality [37] and betweenness centrality [38]) have been proposed by sociologists to determine the "centrality" of a node in a social network. Likewise, in the area of Web graphs, computer scientists have proposed and used several algorithms such as HITS [39] and PageRank [40] for automatically determining the "importance" of Web pages.

In the current study, for the first time, we utilize the above methods to prioritize disease candidate genes by estimating their relative importance in the PPIN to the disease-related genes. Specifically, we determine the optimal parameter values in the methods and record the corresponding performance. The first algorithm that we use is based on White and Smyth's PageRank algorithm. White and Smyth [41] proposed a general framework and a set of algorithms under the framework to measure the relative importance in networks. The first method is an extension of the original PageRank algorithm and is called PageRank with Priors. It mimics the random surfer model wherein a random Internet surfer starts from one of a set of root nodes, R, and follows one of the links randomly in each step. In this process, the surfer jumps back to the root nodes at probability β, thus restarting the whole process. Intuitively, the PageRank with Priors algorithm generates a score that is proportional to the probability of reaching any node in the Web surfing process. This score indicates or measures the relative "closeness" or importance to the root nodes. The second algorithm, named HITS with Priors, is an extension of HITS (Hyperlink-Induced Topic Search), which is a link analysis algorithm developed by Jon Kleinberg to rate Web pages. It determines two values for a page: its authority, which estimates the value of the content of the page, and its hub value, which estimates the value of its links to other pages [42]. In the Web surfing model, the surfer still starts from one of the root nodes. In the odd steps he/she can either follow a random "out-link" or jump back to a root node, and in the even steps he/she can instead follow an "in-link" or jump back to a root node. Similar to the PageRank with Priors, HITS with Priors also estimates the relative probability of reaching a node in the network. The third algorithm we use is the K-Step Markov method. In a similar Web surfing scenario, this method mimics a surfer who starts with one of the root nodes. The surfer follows a random link in each step, but he/she return to the root node after K steps and restarts surfing.

## Results

### Human protein interaction network

The human protein-protein interactions were extracted from the NCBI Entrez Gene FTP site [43] and contained 8340 nodes or vertices (corresponding to 8340 unique genes/proteins) and 27250 edges (corresponding to 27250 unique interactions). This compilation is based on three interaction databases, namely, BIND (2389 genes and 4054 interactions) [44], BioGRID (7683 genes, 23205 interactions) [45], and HPRD (6594 genes and 22802 interactions) [46] (See Additional File 1 for the overlap among these three resources). Although these literature-based or literature-curated interactions are more subjective to research bias, they are less prone to errors. Analysis of this complete human protein interaction network using "NetworkAnalyzer" [47] in Cytoscape [48] revealed 120 connected components. The largest of these has 8075 genes. The remaining 265 genes are separated into 119 smaller components or sub networks of size two to five nodes or genes. Since majority of these smaller sub-networks contain only two genes, we reasoned that it might not be of interest to check the distribution of the disease genes among them.

### Evaluation of PPIN for gene prioritization

**Figure 1:**
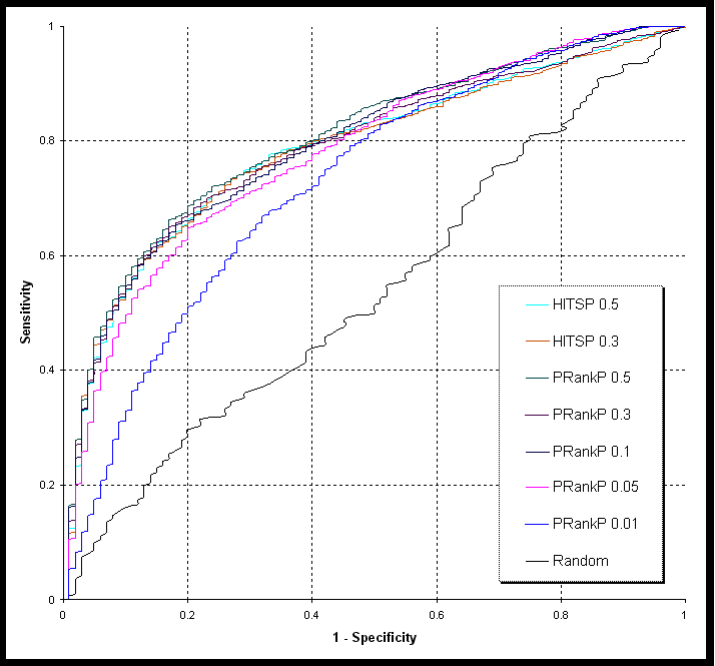
**ROC curves from cross validations**. This figure shows the representative ROC curves using PageRank with Priors with back probability 0.01, 0.05, 0.1, 0.3 and 0.5, and HITS with Priors with back probability 0.3 and 0.5. The random curve was derived from prioritization of the random training set using the PageRank with Prior method with back probability 0.3.

**Figure 2:**
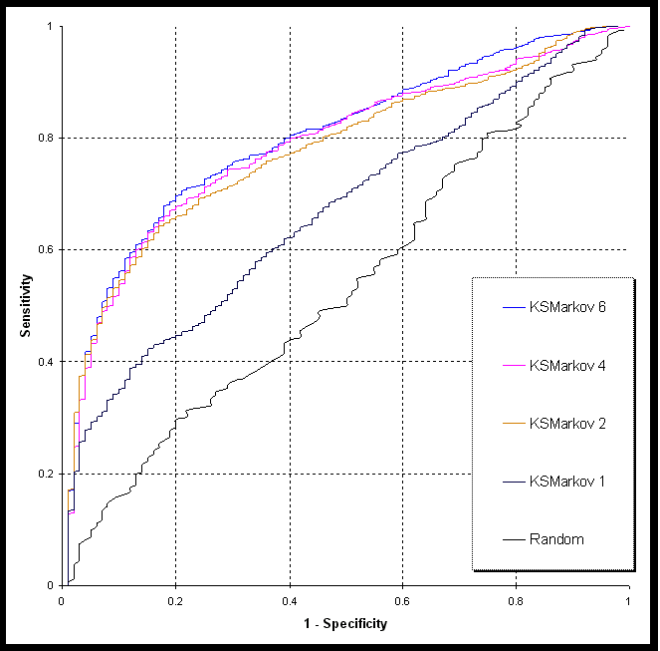
**ROC curves from cross validations**. This figure shows the representative ROC curves using the K-Step Markov method with K = 1, 2, 4, and 6. The random curve was derived from prioritization of the random training set using the PageRank with Prior method with back probability 0.3.

We used the same training data, from our previous study [6], comprising 19 diseases with 693 associated genes. Of these, 589 genes were used in the cross validation because the remaining 104 genes do not have any known protein-protein interactions (see Additional File 2). The random training dataset, used as a control, was built with 19 random gene lists, with each list comprising 31–38 genes. We used three methods, namely, K-Step Markov (KSMarkov), PageRank with Priors (PRankP), and HITS with Priors (HITSP), to prioritize the disease gene with different parameter values. The random genes were prioritized using PRankP with back probability set to 0.3. The ROC curves of representative cross validation results are shown in the figures 1 and 2.

**Figure 3:**
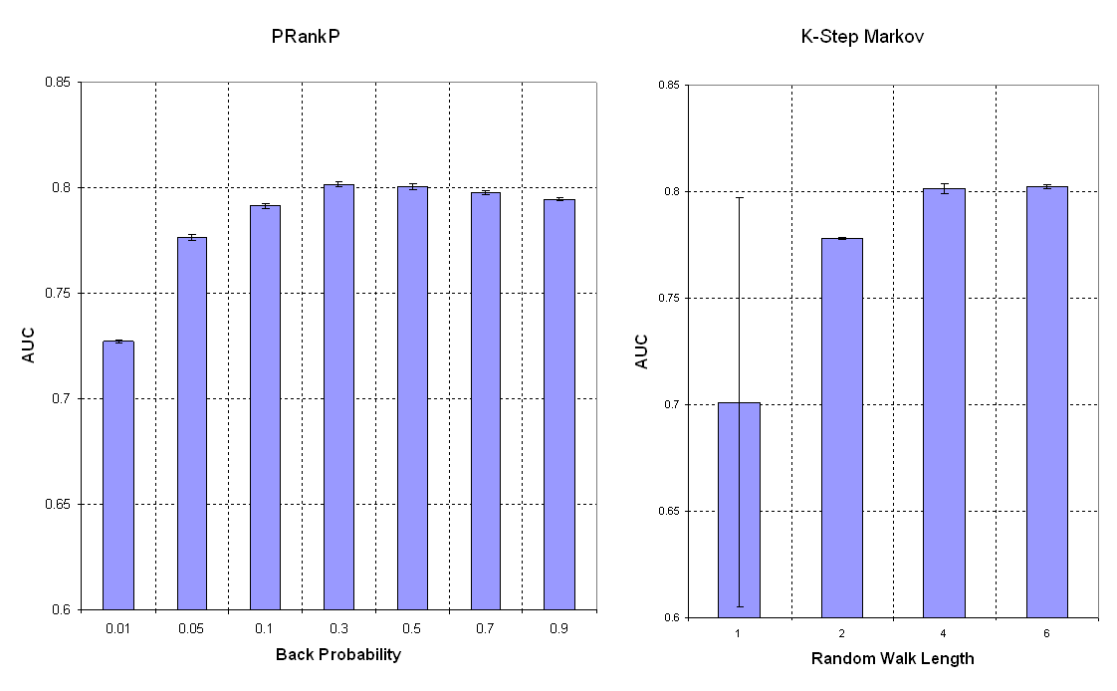
**Plots of AUC with different parameter values**. The left panel shows the AUC values of PageRank with Priors with back probability varied from 0.01 to 0.9. The right panel shows the AUC values of the K-Step Markov method with random walk length varied from 1 to 6. The vertical bars indicate the standard deviations.

Based on our results, we observed that in terms of performance, HITSP was similar to PRankP under different back probability values. Therefore, only PRankP was tested for extreme back probability values such as 0.01 and 0.05. The 13 different test conditions (PRankP with back probability 0.01, 0.05, 0.1, 0.3, 0.5, 0.7, 0.9; KSMarkov with k = 1, 2, 4, 6; and HITSP with Priors with back probability 0.3 and 0.5) along with the AUC values from each validation run are listed in Table 1. Each of the methods, with the same parameter settings, was repeated 5 times. The performance values derived from each of the methods with respect to a particular parameter value are summarized in Table 2. The plots of AUC with different parameter values are shown in figure 3. The best performance of each method was selected, namely, PRankP and HITSP with back probability 0.3 and KSMarkov with K = 4, for Analysis of Variance (ANOVA). The *p value *of 0.5585 suggests that there is no significant difference among the best performance of the three methods.

**Table 1:** AUC values from each cross validation run.

**Test Type**	**Test ID**	**AUC**	**Test ID**	**AUC**	**Test ID**	**AUC**	**Test ID**	**AUC**	**Test ID**	**AUC**
k1	1	0.66	2	0.66	3	0.87	4	0.66	5	0.66

k2	6	0.78	7	0.78	8	0.78	9	0.78	10	0.78

k4	11	0.8	12	0.8	13	0.8	14	0.8	15	0.8

k6	16	0.8	17	0.8	18	0.8	19	0.8	20	0.8

h3	21	0.8	22	0.8	23	0.8	24	0.8	25	0.8

h5	26	0.8	27	0.8	28	0.8	29	0.8	30	0.8

p01	36	0.73	37	0.73	38	0.73	39	0.73	40	0.73

p05	31	0.78	32	0.78	33	0.78	34	0.78	35	0.78

p1	41	0.79	42	0.79	43	0.79	44	0.79	45	0.79

p3	46	0.8	47	0.8	48	0.8	49	0.8	50	0.8

p5	51	0.8	52	0.8	53	0.8	54	0.8	55	0.8

p7	56	0.8	57	0.8	58	0.8	59	0.8	60	0.8

p9	61	0.79	62	0.8	63	0.79	64	0.8	65	0.8

Column "Test Type" indicates the method and parameter settings of the test. p01 through p9 stand for PageRank with Priors with back probability 0.01 to 0.9, respectively; k1, k2, k4 and k6 represent K-Step Markov with K = 1, 2, 4 and 6, accordingly; h3 and h5 are HITS with Priors with back probability 0.3 and 0.5, respectively. There were 13 test conditions, each repeated 5 times.

**Table 2:** Mean and standard deviation (SD) of AUC values with 13 different cross validation conditions.

**Method**	**Parameter**	**Mean of AUC**	**SD of AUC**
PageRank with Priors	Back probability = 0.01	0.73	0.0008

PageRank with Priors	Back probability = 0.05	0.78	0.0015

PageRank with Priors	Back probability = 0.1	0.8	0.0013

PageRank with Priors	**Back probability = 0.3**	**0.8**	**0.0011**

PageRank with Priors	Back probability = 0.5	0.8	0.0015

PageRank with Priors	Back probability = 0.7	0.8	0.0009

PageRank with Priors	Back probability = 0.9	0.79	0.0007

K-Step Markov	K = 1	0.7	0.096

K-Step Markov	K = 2	0.78	0.0005

K-Step Markov	K = 4	0.8	0.0024

K-Step Markov	**K = 6**	**0.8**	**0.0009**

HITS with Priors	**Back probability = 0.3**	**0.8**	**0.0009**

HITS with Priors	Back probability = 0.5	0.8	0.0004

Highlighted rows correspond to the best parameter value of each method.

### Cardiac septal defect candidate gene prioritization

Mining the "clinical synopsis" and "allelic variant sections" of NCBI's OMIM (Online Mendelian Inheritance in Man) database [49], we extracted 166 OMIM records that had the terms "atrial septal defect" OR "ASD" OR "ventricular septal defect" OR "VSD" occurring in the text. There were 81 genes mapping to these records (see Additional File 3 for a list of OMIM records and the corresponding genes associated with cardiac septal defects). These 81 genes were used as the training set. Mining the human protein interactome [43] (see Methods) we extracted the 479 immediate interactants (level 1) of these training 81 genes (Additional File 3). We then sought to rank or prioritize these genes using both integrative functional annotation-based methods and PPIN-based methods using our ToppGene server [6]. There was an overlap of 48 genes which were removed leaving 431 genes for ranking. We call this as the test set for cardiac septal defect.

**Figure 4:**
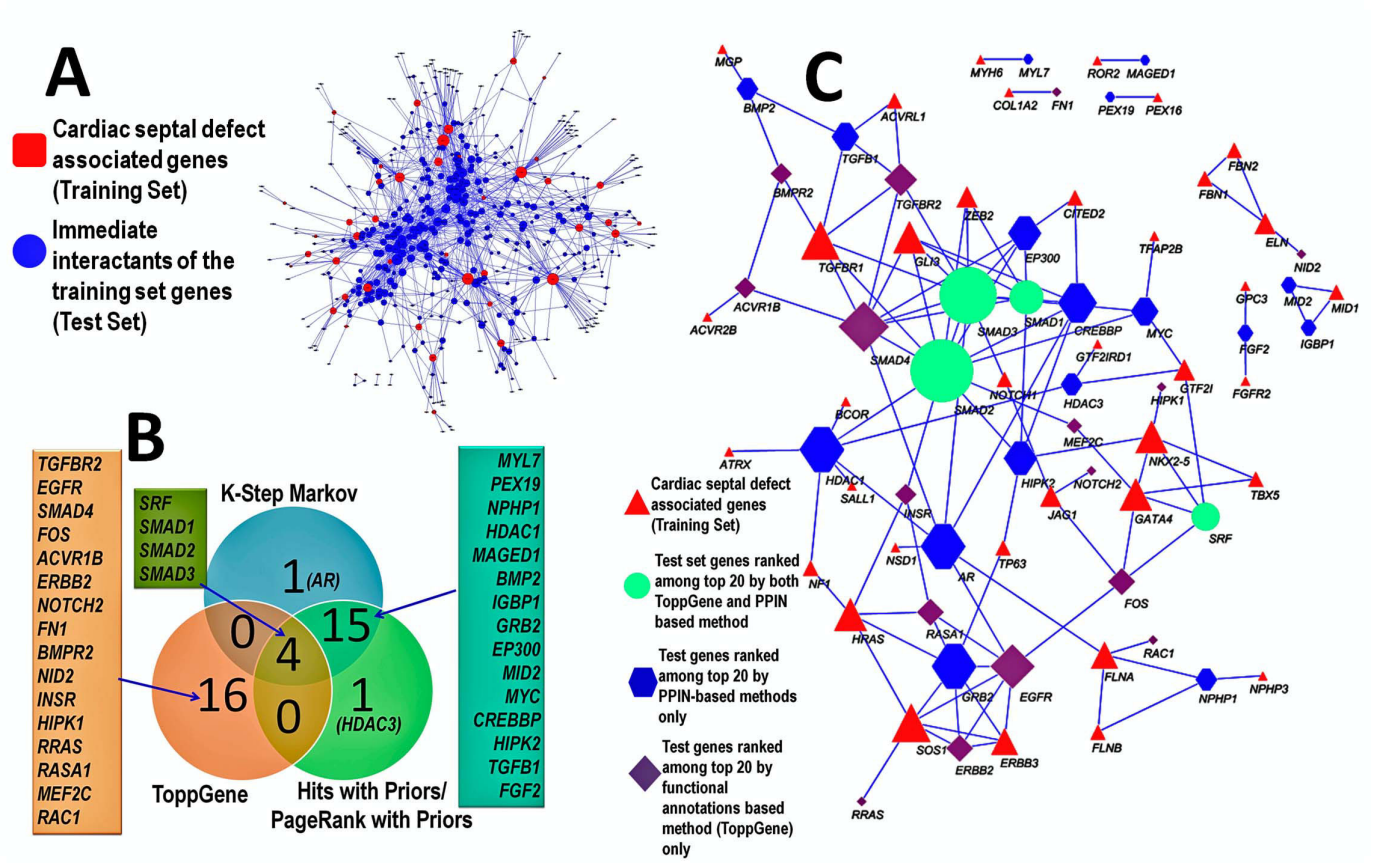
**Prioritized candidate genes of cardiac septal defects using both functional annotation- and PPIN- based methods**. Panel A shows the sub-network of heart septal defect related genes comprising (i) genes associated with OMIM diseases that have the phenotype of cardiac septal defect (Training set of genes for cardiac septal defect) and their immediate interactants (Test set genes). The size of the nodes is proportional to the degree (number of edges). Panel B shows the intersection among the top 20 ranked cardiac septal defect candidate genes using functional annotation- and PPIN- based methods. Functional annotation-based prioritization was done using ToppGene server. For PPIN-based methods K-Step Markov, Hits with Priors, and PageRank with Priors was used. Panel C shows the top 20 ranked cardiac septal defect genes (generated using PPIN- and functional annotation- based methods) along with their connectivity to training set genes (based on protein-protein interactions).

Among the top 20 ranked genes (Table 3), 4 genes (*SRF*, *SMAD1*, *SMAD2*, *SMAD3*) were common to all the methods (Figure 4). Analyzing the results we observed that the performance of both the approaches (functional annotation- versus PPIN- based methods) was comparable. For instance, among the top 20 ranked genes using functional annotations, 15 genes were reported to be previously associated with cardiac development or malformation (indicated with an asterisk in the Table 3). Six (*INSR, ERBB2, NOTCH2, BMPR2, TGFBR2 *and *SRF) *of these top 20 have been previously reported to be associated with cardiac septal defects. In case of PPIN-based methods, there were 14 genes previously associated with either cardiac development or abnormalities. Of these, 3 genes (*SRF, EP300*, *and CREBBP*) have been associated with cardiac septal defect. The genes *EP300 *and *CREBBP *have been ranked 11/431 and 15/431 using PPIN-based methods while the rankings were 41 and 40 respectively using ToppGene. Interestingly, truncated CBP protein (gene *CREBBP*) leads to classical Rubinstein-Taybi syndrome phenotypes in mice characterized by atrial and ventricular septal defects [50]. Likewise, mouse embryos lacking p300 protein (gene *EP300*) show ventricular septal defects [51]. The higher ranking of *EP300 *and *CREBBP *in PPIN-based method is because of their direct interactions with training set gene (*CITED2*). Previous studies have reported that the paralogous genes *EP300 *and *CREBBP *co-activate *TFAP2A *in the presence of *CITED2 *[52]. Similarly, *MYL7 *is ranked first in PPIN-based prioritization while it is ranked 122 in functional annotation-based prioritization methods. The higher ranking in the former is because *MYL7 *has only one known interactant (*MYH6*), mutation of which is associated with cardiac septal defects [53]. Another noteworthy example is *BMP2*, ranked 6/431 by PPIN-based method while the ToppGene rank was 32/431. On the other hand there were examples of potential candidate genes which the PPIN-based prioritization methods ranked low while ToppGene ranked them higher. For instance, *ERBB2 *was ranked 112/431 by PPIN-based method while functional annotation-based gene prioritization (ToppGene) ranked it as eight. Mice with a ventricular-restricted deletion of *Erbb2 *show ventricular septal defect (VSD) [54,55] suggesting that the human ortholog *ERBB2 *could be a potential candidate gene for VSD. Thus, while integrative functional annotation-based methods are superior in prioritizing disease candidate genes, PPIN-based methods certainly have their own advantages. We, therefore, hypothesize that a combined functional annotations- and PPIN- based methods are more effective in identifying and ranking of disease candidate genes. The rankings of all the test (431) genes using different methods (PPIN-based, ToppGene and ENDEAVOUR) are included in the Additional Files 4 and 5. Further, given the continued incomplete annotation coverage of human genes (see Table 4 for a summary of functional annotation coverage of human interactome genes and Additional File 6 for a gene-wise breakdown of all annotations and protein interactions), PPIN-based prioritization is a viable option.

**Table 3:** Cardiac septal defect candidate gene prioritization.

**Rank**	**Integrative functional annotation based ranking (using ToppGene)**	**PPIN-based ranking (K-Step Markov)**	**PPIN-based ranking (Hits with Priors)**	**PPIN-based ranking (PageRank with Priors)**
1	TGFBR2*^#^	MYL7*	MYL7*	MYL7*

2	EGFR*	*PEX19*	*PEX19*	*PEX19*

3	SMAD4*	*NPHP1*	*NPHP1*	*NPHP1*

4	SRF*^#^	HDAC1*	*MAGED1*	*MAGED1*

5	*FOS*	*MAGED1*	BMP2*	BMP2*

6	*ACVR1B*	BMP2*	HDAC1*	HDAC1*

7	SMAD2*	SRF*^#^	*IGBP1*	*IGBP1*

8	ERBB2*^#^	*IGBP1*	*MID2*	*MID2*

9	NOTCH2*^#^	GRB2*	SMAD3*	SMAD3*

10	FN1*	SMAD3*	SRF*^#^	SRF*^#^

11	BMPR2*^#^	EP300*^#^	GRB2*	GRB2*

12	*NID2*	*MID2*	MYC*	MYC*

13	SMAD1*	MYC*	*HIPK2*	*HIPK2*

14	SMAD3*	SMAD2*	FGF2*	FGF2*

15	INSR*^#^	CREBBP*^#^	SMAD2*	SMAD2*

16	*HIPK1*	SMAD1*	SMAD1*	SMAD1*

17	*RRAS*	*HIPK2*	*HDAC3*	*HDAC3*

18	RASA1*	TGFB1*	EP300*^#^	EP300*^#^

19	MEF2C*	FGF2*	CREBBP*^#^	CREBBP*^#^

20	RAC1*	AR*	TGFB1*	TGFB1*

The cardiac septal defect sub-network was created using known cardiac septal defect genes (from OMIM) and their immediate interactants, and was prioritized using functional annotation and PPIN based methods. Functional annotation based prioritization was done using ToppGene server. The PPIN based rankings were obtained using 3 methods: K step Markov, Hits with Priors, and PageRank with Priors. The highlighted genes are those occurring in all of the prioritized top 20 genes generated using different methodologies. Note that the Hits with Priors and PageRank with Priors gave identical results (see Additional Files 3, 4 and 5 for the list of genes and prioritization results). The genes marked with * are associated with abnormal heart morphology (ToppGene: 15/20; K-Step Markov: 14/20; and Hits with Priors and PageRank with Priors: 13/20) while those marked with # have been reported to be associated with cardiac septal defects (6/20 and 3/20 in ToppGene and PPIN prioritized top 20 candidate genes for cardiac septal defects).

**Table 4:** Summary of functional annotation coverage of human interactome genes.

***Interactome genes with 3 annotations (2440)***	
GO + MP + Pathways	2440

***Interactome genes with any 2 annotations (2866)***	

GO + Pathways	1630

GO+MP	1232

MP + Pathways	4

***Interactome genes with only 1 annotation (2505)***	

GO only	2448

MP only	10

Pathways only	47

***Interactome genes with no annotations (223)***	223

***Total Interactome genes***	**8034**

About 1/3^rd ^(2505/8034) genes of the interactome are sparsely annotated (GO – Gene Ontology; MP – Mammalian Phenotype; and Pathway annotations).

## Discussion and Conclusion

Our current study, based on the observation that biological networks share many properties with Web and social networks, is an attempt to extend the successful graph analysis-based algorithms from computer science research to tackle the disease gene prioritization problem. Literature-based and manually curated protein interactions were used to form the base network, and extended versions of the PageRank algorithm and HITS algorithm, as well as the K-Step Markov method, were applied to prioritize disease candidate genes in a training-test schema. For each prioritization, a list of known disease-related genes was used as a training set ("seeds"), and the genes in the test list (candidate genes) were ranked. To evaluate and compare the performance of the methods, a large-scale cross validation was performed. A total of 13 conditions with three algorithms and different parameter settings were tested, each repeated five times. Rank-based ROC curves were plotted, and AUC values were used to quantitatively measure the performance.

Based on our results, we draw the following conclusions: First, under appropriate settings, for example, a back probability of 0.3 for PageRank with Priors and HITS with Priors, and walk length 6 for K-Step Markov method, the three methods achieved the same AUC value and hence similar performance. This suggests that based on the current knowledge of protein-protein interaction networks, even other similar or related methods (e.g., ranking of nodes in an unweighted graph) under the same framework might yield similar results.

Second, the value of back probability in PageRank with Priors and HITS with Priors can be of broad range (e.g., 0.1 to 0.9) and still result in relatively stable performance. However, when the back probability was set to very low (e.g., 0.01), the performance dropped significantly. This is expected because in both the methods (see equations 3 and 4 under Methods), as the back probability reaches 0, the bias toward the "seeds" is eliminated. PageRank/HITS with Priors are same as the original PageRank/HITS algorithm; therefore, the prioritization toward the selected "seeds" fails. The performance of the K-Step Markov method, on the other hand, decreased significantly when the length of random walk K was small (e.g. K = 1). Under this condition, the K-Step Markov method calculates the probability to spend time on each protein from the seeds with a random walk of length 1. The proteins that are not directly interacting with "seeds" will therefore never be reached and scored 0. This suggests that if a true disease candidate gene is not directly interacting with the "seeds", it will be ignored when K is 1. The method converged to the best performance when K was 4. Any further increase in the random walk length did not improve the performance. This can be attributed to the fact that the average shortest path length in the PPIN was only about 4.5.

Third, the overall performance of candidate gene prioritizations based exclusively on protein networks is comparable to functional annotation-based methods [6] since they were all tested using the same cross validation. The AUC value of functional annotation based method, ToppGene [6], was 0.916, and the best AUC value of network-based methods (from the current study) was 0.801. This shows that network-based methods are generally not as effective as the integrated functional annotation-based methods for disease candidate gene prioritization. For a more accurate comparison, we compared PPIN-based methods to the individual functional annotation features used in our previous study [6]. Surprisingly, we found that network-based methods are better than all annotations (see [6] for details). We therefore conclude that PPINs can be a potentially good feature for disease candidate gene prioritization irrespective of whether the genes have other functional annotations or not. Based on our findings that in one-to-one comparison PPIN-based candidate gene prioritization performed better than all other gene features or annotations, we hypothesize that PPINs can be a potentially good feature for disease candidate gene prioritization, especially when the genes lack all other functional annotations or are sparsely annotated

Network-based prioritization methods, however, have certain limitations. Just like functional annotation-based methods, the performance depends on the quality of interaction data. It is an acknowledged fact that the current human protein interactome suffers with incompleteness and unreliability with missing interactions and false positives. To make reliable candidate gene prioritization – based either on functional annotations or PPINs – we must have reasonably complete datasets that accurately represent the interactions and annotations in the genome and proteome. However, as the quality of these annotations and interactions improves the confidence in candidate gene prioritization approaches based on them will also improve. Certainly, our approach can be improved methodology-wise in the following directions. First, the algorithms used in our current study were originally developed to identify "important" nodes in networks. Although we used extended versions of these algorithms to prioritize nodes to selected "seeds," there still could be a bias toward hubs. Additionally, since these approaches were designed for Web and general networks, there is definitely scope for additional modifications to make them fit better with biological networks (e.g., using weighted nodes (genes or proteins) or edges (interactions)). As future extension, apart from considering weighted nodes and edges, we plan to integrate our method with other methods (e.g., combining results from functional annotation-based methods and expression profiles with network-based approaches). It is expected that using both functional annotations and PPIN-based topological parameters may better facilitate the discovery and prioritization of disease genes.

## Methods

### Human protein interaction datasets

The human protein interaction dataset (file "interactions.gz"), a compilation of PPIs from BIND [44], BioGRID [45], and HPRD [46], was downloaded from NCBI Entrez Gene FTP site [43]. All of these interactions are derived from large-scale experiments and curated manually. For example, all interactions in BIND are experimentally validated and published in at least one peer-reviewed journal; interactions in BioGRID are entirely derived from manual literature curation, just as in HPRD.

### Prioritization methods

In the current study, the protein interaction network is represented as an unweighted, undirected simple graph, G, where proteins (genes) are nodes and interactions are edges. The set of all the proteins in the network is denoted as V and all the interactions as E. The set of known disease genes (also called the seeds) is denoted as R. The prioritization approaches are based on the methods of White and Smyth [41], whose general framework, consisting of four successive problem formulations, each building on the next, defines the approach to ranking nodes in an unweighted digraph G(V, E):

*1. Relative importance of a node t with respect to a root node r*: Given G and r and t, where r and t are both nodes in G and r is the root, compute the "importance" of t with respect to r. This importance is denoted as I(t|r), a non-negative quantity.

*2. Rank of importance of a set of nodes T with respect to a root node r*: Given G and a root node r in G, rank all vertices in T, a subset of vertices in G. For each node t in T, the value of I(t|r) can be computed. Then the nodes can be ranked so that the largest values correspond to the highest importance.

*3. Rank of importance of a set of nodes T with respect to a set of root nodes R*: Given G and a set of root node R in G, rank all vertices in T, a subset of vertices in G. The importance of node t to R is defined as the average sum of importance of t to each node in R:

(1) I(t|R) = (1/|R|)(sum(I(t|r)).

*4. Given G, rank all nodes*: This is a special case where R = T = V.

Based on White and Smyth's framework, the solution to problem 3 is what is needed in this study. To recap it in the context of disease gene prioritization, the problem is to prioritize a set of genes in the network based on their importance to a set of root genes (e.g., genes known to be associated with a disease). The importance of a gene to the set of root genes is just the average sum of the importance of it to each individual root gene. Although this framework was proposed for directed networks, it can also be applied to the undirected networks because the latter is just a special case of the former. In this study, the undirected protein interaction network was converted to an equivalent directed network, when necessary.

With the problem formulation defined, the key of the solution is to find I(t|r), the importance of node t with respect to a root node r. For this, we use three algorithms from White and Smyth [41]: a) PageRank with Priors, b) HITS with Priors, and c) K-step Markov.

The PageRank with Priors method is an extension of the original PageRank algorithm. The iterative stationary probability equation is:

(2) $$\pi {\left(v\right)}^{\left(i+1\right)}=(1-\beta )\left(\sum_{u=1}^{d_{in}(v)}p(v|u)\pi^{\left(i\right)}(u)\right)+\beta p_{v}$$

In this equation, p_v _represents the "prior bias". p_v _= 1/|R| for v in R, the root node set; p_v _= 0 otherwise. β, empirically defined on [0, 1], represents a "back probability." d_in_(v) is the in-degree of v. p(v|u) is the probability of arriving vertex v from u. With the surfing model described earlier taken in consideration, "Prior bias" represents the probability to start with a particular node. In this case, all root nodes are considered equally important; therefore prior bias is 1/|R| for all root nodes. The prior bias in case of non-root nodes is set to 0 to eliminate the probability of starting with a non-root node. The "back probability" represents the probability to jump back to the root node in each step.

The HITS with Priors is an extension of the original HITS algorithm. The iterative equations are defined as:

(3) $$\begin{array}{c} a^{\left(i+1\right)}(v)=(1-\beta )\left(\sum_{u=1}^{d_{in}(v)}\frac{h^{\left(t\right)}(u)}{H^{\left(i\right)}}\right)+\beta p_{v}\\ h^{\left(i+1\right)}(v)=(1-\beta )\left(\sum_{u=1}^{d_{out}(v)}\frac{a^{\left(t\right)}(u)}{A^{\left(i\right)}}\right)+\beta p_{v} \end{array}$$

where d_in_(v) and d_out_(v) are the in-degree and out-degree of v, respectively, and H^(i) ^and A^(i) ^are defined as:

(4) $$\begin{array}{c} H^{\left(i\right)}=\sum_{v=1}^{\left|V\right|}\sum_{u=1}^{d_{in}(v)}h^{\left(i\right)}(u)\\ A^{\left(i\right)}=\sum_{v=1}^{\left|V\right|}\sum_{u=1}^{d_{out}(v)}a^{\left(i\right)}(u) \end{array}$$

For definitions of prior bias p_v _and "back probability" β refer to the earlier sections under PageRank with Priors. The authority score is set as the importance of the node.

The K-Step Markov approach computes the relative probability that the system will spend time at any particular node given that it starts in a set of roots R and ends after K steps. According to White and Smyth [41], the value of K controls the relative trade-off between a distribution "biased" toward R, and when K gets larger the steady-state distribution will converge to the PageRank result. The equation to compute the K-Step Markov importance is:

(5) *I*(*t*|*R*) = [Ap_*R *_+ A^2^p_*R*_...A^*K*^p_*R*_]_*t*_

where A is the transition probability matrix of size n × n, p_R _is an n × 1 vector of initial probabilities for the root set R, and I(t|R) is the t-th entry in this sum vector.

For additional details of the methods, the readers are referred to the original paper by White and Smyth [41].

### PPIN analysis and derivation of topological parameters

The basic network statistics and topological parameters were derived using NetworkAnalyzer [47]. NetworkAnalyzer is a *JAVA *plug-in for Cytoscape [48], a software platform for the analysis and visualization of molecular interaction networks. The version of Cytoscape was 2.5.2 and NetworkAnalyzer was 2.5.1.

The implementation of the prioritization methods, PageRank with Priors, HITS with Priors, and the K-Step Markov approach are all available in the JUNG (*JAVA *Universal Network/Graph) [56] framework. It is a *JAVA *package that provides a common and extendible language for the modeling, analysis, and visualization of data that can be represented as a graph or network. Version 2.0 was used and integrated with other in-house programs through APIs to perform all the required functions. Further details of JUNG can be obtained from the web site [56].

### Evaluation of prioritization methods

Cross-validations to test the performance of the prioritization methods were done as described earlier [6]. Briefly, 19 diseases from OMIM [57] and GAD [58] were used as training sets. For each disease, the associated genes (with the one under test removed) were used as "seeds" and leave-one-out random cross-validation was performed. Random sets of genes were used as the control training sets. The rank-based sensitivity and specificity followed the previous definitions. ROC curves were plotted to visualize the performance with AUC values as quantitative measures. For further details refer to our previous publication [6].

All of the three node ranking methods require pre-determined parameters. For PageRank with Priors and HITS with Priors, the "back probability" is needed. It represents the bias toward the seeds, and the recommended value, according to White and Smyth [41], is 0.3. For the K-Step Markov approach, the only parameter is the length of the random walk, which controls the relative trade-off between a distribution "biased" toward the "seeds" and the steady-state distribution, which is independent of the "seeds." As K gets bigger, the final state is moving toward the steady state. The recommended K value was 6. In order to evaluate the effect of different values of the parameters on the performance, different values of parameters were used in the cross-validations and a test of each parameter setting was repeated five times to estimate the mean and standard deviation. Comparison of the performance of each of the three methods was done through analysis of variance.

### Cardiac septal defect gene network

To obtain a list of all diseases that have a phenotype cardiac septal defect, we queried the "Clinical Synopsis" and "Allelic Variants" sections of OMIM database with the terms "atrial septal defect" or "ventricular septal defect" or "ASD" or "VSD". We then downloaded the associated genes (Training set) and their immediate interactants (Test set) based on PPIN. The test genes were then ranked using (a) functional annotation-based prioritization (ToppGene server [6]); and (b) PPIN-based ranking (as described earlier). The network view of the top 20 ranked genes along with their interactions with the training set genes was generated using Cytoscape (version 2.6.1) and the plug-in "NetworkAnalyzer" [47,48].

## Authors' contributions

JC, BA, and AJ conceived the study design, which was coordinated by AJ. JC designed and implemented the gene prioritization algorithms and along with AJ participated in the analysis and interpretation of results. JC and AJ drafted the manuscript. All the authors have read and approved the final manuscript.

## Supplementary Material

Additional file 1 **Venn diagrams of unique genes and interactions from the three (BIND, BioGRID, and HPRD) PPIN data source.** Click here for file

Additional file 2 **Training set data used for evaluation of PPIN in disease candidate gene prioritization, comprising 19 diseases with 693 associated genes.** Of these, 589 genes were used in the cross validation because the rest (104 genes) had no reported interactions. Click here for file

Additional file 3 **Cardiac septal defect associated OMIM records, genes and their immediate interactants (based on protein-protein interactions).** Click here for file

Additional file 4 **Prioritized candidate genes for cardiac septal defects using PPIN- and functional annotation- based methods.** Click here for file

Additional file 5 **Prioritized candidate genes for cardiac septal defects using three PPIN-based methods and two functional annotation- based methods (ToppGene and ENDEAVOUR).** Click here for file

Additional file 6 **Annotation and PPIN coverage of the human genes.** Click here for file
