# Correction: Disease candidate gene identification and prioritization using protein interaction networks

**DOI:** 10.1186/1471-2105-10-406

**Published:** 2009-12-09

**Authors:** Jing Chen, Bruce J Aronow, Anil G Jegga

**Affiliations:** 1 Division of Biomedical Informatics, Cincinnati Children's Hospital Medical Center, Cincinnati, USA; 2 Department of Biomedical Engineering, University of Cincinnati, Cincinnati, USA; 3 Department of Pediatrics, University of Cincinnati College of Medicine, Cincinnati, USA

## Text

After the publication of this work [1], we became aware of the fact that we inadvertently failed to include the work of Chen et al. [2] when listing previous studies that use PPIs to prioritize disease candidate genes. In a pioneering study [2], Chen et al. used a initial gene list for Alzheimer's from the OMIM database, expanded it based on protein interactions, and proposed a scoring function to identify other Alzheimer's disease causal genes based on graph-connectedness.
