# Supplementary material for: Disease candidate gene identification and prioritization using protein interaction networks
Source: BMC Bioinformatics. 2009 Feb 27;10:73. doi: 10.1186/1471-2105-10-73 (PMC2657789; doi:10.1186/1471-2105-10-73)

Additional File 1: Venn diagrams of unique genes and interactions from the three (BIND, BioGRID, and HPRD) PPIN data sources.

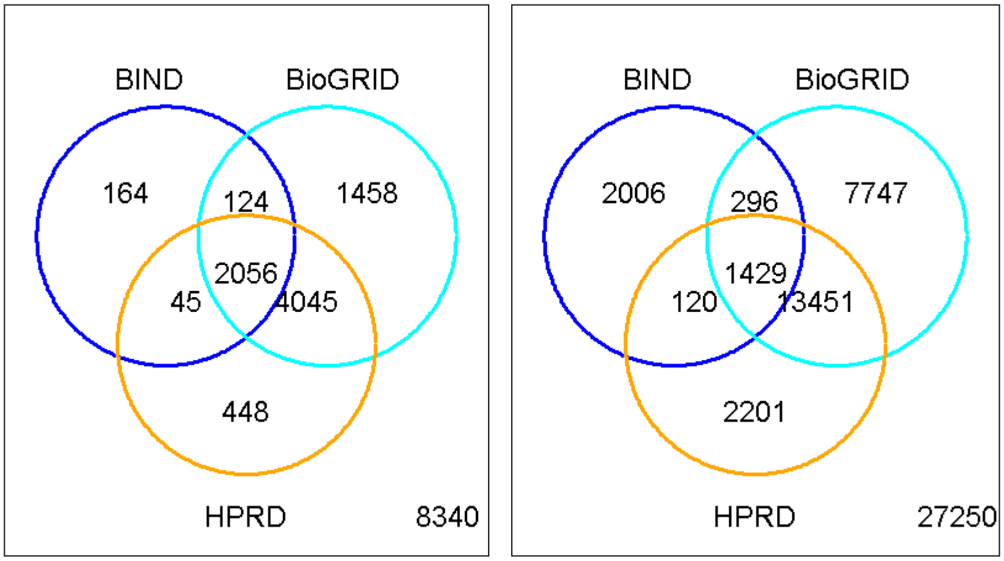

Supplement: Additional file 1 — Venn diagrams of unique genes and interactions from the three (BIND, BioGRID, and HPRD) PPIN data source. [file 1471-2105-10-73-S1.pdf]
