# Supplementary material for: Disease candidate gene identification and prioritization using protein interaction networks
Source: BMC Bioinformatics. 2009 Feb 27;10:73. doi: 10.1186/1471-2105-10-73 (PMC2657789; doi:10.1186/1471-2105-10-73)
Supplement: Additional file 2 — Training set data used for evaluation of PPIN in disease candidate gene prioritization, comprising 19 diseases with 693 associated genes. Of these, 589 genes were used in the cross validation because the rest (104 genes) had no reported interactions. [file 1471-2105-10-73-S2.pdf]

Disease name	"Target" gene ID	“Target” gene symbol	"Target" gene name	"Target" gene in PPIN?
**Ischaemic stroke**	1636	ACE	angiotensin I converting enzyme (peptidyl-dipeptidase A) 1	Yes
	241	ALOX5AP	arachidonate 5-lipoxygenase-activating protein	Yes
	335	APOA1	apolipoprotein A-I	Yes
	348	APOE	apolipoprotein E	Yes
	11132	CAPN10	calpain 10	No
	6347	CCL2	chemokine (C-C motif) ligand 2	Yes
	929	CD14	CD14 molecule	Yes
	1401	CRP	C-reactive protein, pentraxin-related	Yes
	2053	EPHX2	epoxide hydrolase 2, cytoplasmic	No
	2161	F12	coagulation factor XII (Hageman factor)	Yes
	2162	F13A1	coagulation factor XIII, A1 polypeptide	Yes
	2147	F2	coagulation factor II (thrombin)	Yes
	2153	F5	coagulation factor V (proaccelerin, labile factor)	Yes
	2155	F7	coagulation factor VII (serum prothrombin conversion accelerator)	Yes
	2243	FGA	fibrinogen alpha chain	Yes
	2244	FGB	fibrinogen beta chain	Yes
	2811	GP1BA	glycoprotein Ib (platelet), alpha polypeptide	Yes
	3106	HLA-B	major histocompatibility complex, class I, B	Yes
	3119	HLA-DQB1	major histocompatibility complex, class II, DQ beta 1	Yes
	3122	HLA-DRA	major histocompatibility complex, class II, DR alpha	Yes
	3123	HLA-DRB1	major histocompatibility complex, class II, DR beta 1	Yes
	3383	ICAM1	intercellular adhesion molecule 1 (CD54), human rhinovirus receptor	Yes
	3553	IL1B	interleukin 1, beta	Yes
	3569	IL6	interleukin 6 (interferon, beta 2)	Yes
	3673	ITGA2	integrin, alpha 2 (CD49B, alpha 2 subunit of VLA-2 receptor)	Yes
	3674	ITGA2B	integrin, alpha 2b (platelet glycoprotein IIb of IIb/IIIa complex, antigen CD41)	Yes
	3675	ITGA3	integrin, alpha 3 (antigen CD49C, alpha 3 subunit of VLA-3 receptor)	Yes
	3676	ITGA4	integrin, alpha 4 (antigen CD49D, alpha 4 subunit of VLA-4 receptor)	Yes
	3690	ITGB3	integrin, beta 3 (platelet glycoprotein IIIa, antigen CD61)	Yes
	4282	MIF	macrophage migration inhibitory factor (glycosylation-inhibiting factor)	Yes
	4314	MMP3	matrix metallopeptidase 3 (stromelysin 1, progelatinase)	Yes
	4524	MTHFR	5,10-methylenetetrahydrofolate reductase (NADPH)	Yes
	4846	NOS3	nitric oxide synthase 3 (endothelial cell)	Yes
	4883	NPR3	natriuretic peptide receptor C/guanylate cyclase C (atrionatriuretic peptide receptor C)	Yes
	5144	PDE4D	phosphodiesterase 4D, cAMP-specific (phosphodiesterase E3 dunce homolog, Drosophila)	Yes
	5327	PLAT	plasminogen activator, tissue	Yes
	5444	PON1	paraoxonase 1	Yes
	8858	PROZ	protein Z, vitamin K-dependent plasma glycoprotein	No
	6401	SELE	selectin E (endothelial adhesion molecule 1)	Yes
	5265	SERPINA1	serpin peptidase inhibitor, clade A (alpha-1 antiproteinase, antitrypsin), member 1	Yes
	5054	SERPINE1	serpin peptidase inhibitor, clade E (nexin, plasminogen activator inhibitor type 1), member 1	Yes
	6548	SLC9A1	solute carrier family 9 (sodium/hydrogen exchanger), member 1 (antiporter, Na+/H+, amiloride sensitive)	Yes
	7099	TLR4	toll-like receptor 4	Yes
	7124	TNF	tumor necrosis factor (TNF superfamily, member 2)	Yes
**Endometriosis**	196	AHR	aryl hydrocarbon receptor	Yes
	197	AHSG	alpha-2-HS-glycoprotein	Yes
	367	AR	androgen receptor (dihydrotestosterone receptor; testicular feminization; spinal and bulbar muscular atrophy; Kennedy disease)	Yes
	405	ARNT	aryl hydrocarbon receptor nuclear translocator	Yes
	6352	CCL5	chemokine (C-C motif) ligand 5	Yes
	1231	CCR2	chemokine (C-C motif) receptor 2	Yes
	1234	CCR5	chemokine (C-C motif) receptor 5	Yes
	1312	COMT	catechol-O-methyltransferase	Yes
	1493	CTLA4	cytotoxic T-lymphocyte-associated protein 4	Yes
	1586	CYP17A1	cytochrome P450, family 17, subfamily A, polypeptide 1	Yes
	1588	CYP19A1	cytochrome P450, family 19, subfamily A, polypeptide 1	Yes
	1543	CYP1A1	cytochrome P450, family 1, subfamily A, polypeptide 1	Yes
	1545	CYP1B1	cytochrome P450, family 1, subfamily B, polypeptide 1	Yes
	1571	CYP2E1	cytochrome P450, family 2, subfamily E, polypeptide 1	Yes
	2052	EPHX1	epoxide hydrolase 1, microsomal (xenobiotic)	No
	2099	ESR1	estrogen receptor 1	Yes
	2100	ESR2	estrogen receptor 2 (ER beta)	Yes

	355	FAS	Fas (TNF receptor superfamily, member 6)	Yes
	356	FASLG	Fas ligand (TNF superfamily, member 6)	Yes
	2592	GALT	galactose-1-phosphate uridylyltransferase	Yes
	2944	GSTM1	glutathione S-transferase M1	Yes
	2952	GSTT1	glutathione S-transferase theta 1	No
	3105	HLA-A	major histocompatibility complex, class I, A	Yes
	3106	HLA-B	major histocompatibility complex, class I, B	Yes
	3107	HLA-C	major histocompatibility complex, class I, C	Yes
	3115	HLA-DPB1	major histocompatibility complex, class II, DP beta 1	Yes
	3117	HLA-DQA1	major histocompatibility complex, class II, DQ alpha 1	No
	3119	HLA-DQB1	major histocompatibility complex, class II, DQ beta 1	Yes
	3123	HLA-DRB1	major histocompatibility complex, class II, DR beta 1	Yes
	3292	HSD17B1	hydroxysteroid (17-beta) dehydrogenase 1	No
	3383	ICAM1	intercellular adhesion molecule 1 (CD54), human rhinovirus receptor	Yes
	3458	IFNG	interferon, gamma	Yes
	3565	IL4	interleukin 4	Yes
	3569	IL6	interleukin 6 (interferon, beta 2)	Yes
	4312	MMP1	matrix metallopeptidase 1 (interstitial collagenase)	Yes
	4314	MMP3	matrix metallopeptidase 3 (stromelysin 1, progelatinase)	Yes
	4353	MPO	myeloperoxidase	No
	10	NAT2	N-acetyltransferase 2 (arylamine N-acetyltransferase)	No
	8204	NRIP1	nuclear receptor interacting protein 1	Yes
	5241	PGR	progesterone receptor	Yes
	7124	TNF	tumor necrosis factor (TNF superfamily, member 2)	Yes
	7133	TNFRSF1B	tumor necrosis factor receptor superfamily, member 1B	Yes
	7422	VEGFA	vascular endothelial growth factor A	Yes
**Autism**	100	ADA	adenosine deaminase	Yes
	116987	CENTG2	centaurin, gamma 2	Yes
	129446	XIRP2(CMYA3)	xin actin-binding repeat containing 2	No
	1641	DCX	doublecortex; lissencephaly, X-linked (doublecortin)	Yes
	2020	EN2	engrailed homeobox 2	Yes
	2332	FMR1	fragile X mental retardation 1	Yes
	93986	FOXP2	forkhead box P2	Yes
	2477	FRAXA	fragile site, folic acid type, rare, fra(X)(q27.3) A (macroorchidism, mental retardation)	No
	2481	FRAXE	fragile site, folic acid type, rare, fra(X)(q28) E	No
	2482	FRAXF	fragile site, folic acid type, rare, fra(X)(q28) F	No
	2555	GABRA2	gamma-aminobutyric acid (GABA) A receptor, alpha 2	No
	2557	GABRA4	gamma-aminobutyric acid (GABA) A receptor, alpha 4	Yes
	2558	GABRA5	gamma-aminobutyric acid (GABA) A receptor, alpha 5	No
	2562	GABRB3	gamma-aminobutyric acid (GABA) A receptor, beta 3	Yes
	2565	GABRG1	gamma-aminobutyric acid (GABA) A receptor, gamma 1	No
	2567	GABRG3	gamma-aminobutyric acid (GABA) A receptor, gamma 3	Yes
	2739	GLO1	glyoxalase I	No
	2898	GRIK2	glutamate receptor, ionotropic, kainate 2	Yes
	2918	GRM8	glutamate receptor, metabotropic 8	Yes
	2925	GRPR	gastrin-releasing peptide receptor	Yes
	3123	HLA-DRB1	major histocompatibility complex, class II, DR beta 1	Yes
	3198	HOXA1	homeobox A1	Yes
	3265	HRAS	v-Ha-ras Harvey rat sarcoma viral oncogene homolog	Yes
	3356	HTR2A	5-hydroxytryptamine (serotonin) receptor 2A	Yes
	4128	MAOA	monoamine oxidase A	No
	4204	MECP2	methyl CpG binding protein 2 (Rett syndrome)	Yes
	9968	MED12	mediator complex subunit 12	Yes
	4763	NF1	neurofibromin 1 (neurofibromatosis, von Recklinghausen disease, Watson disease)	Yes
	54413	NLGN3	neuroligin 3	Yes
	57502	NLGN4X	neuroligin 4, X-linked	Yes
	4974	OMG	oligodendrocyte myelin glycoprotein	Yes
	5021	OXTR	oxytocin receptor	Yes
	5728	PTEN	phosphatase and tensin homolog (mutated in multiple advanced cancers 1)	Yes
	5803	PTPRZ1	protein tyrosine phosphatase, receptor-type, Z polypeptide 1	Yes
	5649	RELN	reelin	Yes
	5054	SERPINE1	serpin peptidase inhibitor, clade E (nexin, plasminogen activator inhibitor type 1), member 1	Yes
	8604	SLC25A12	solute carrier family 25 (mitochondrial carrier, Aralar), member 12	Yes

	6532	SLC6A4	solute carrier family 6 (neurotransmitter transporter, serotonin), member 4	Yes
	7054	TH	tyrosine hydroxylase	Yes
	121278	TPH2	tryptophan hydroxylase 2	No
	7472	WNT2	wingless-type MMTV integration site family member 2	Yes
**Lymphoma**	1636	ACE	angiotensin I converting enzyme (peptidyl-dipeptidase A) 1	Yes
	9370	ADIPOQ	adiponectin, C1Q and collagen domain containing	Yes
	596	BCL2	B-cell CLL/lymphoma 2	Yes
	604	BCL6	B-cell CLL/lymphoma 6 (zinc finger protein 51)	Yes
	1026	CDKN1A	cyclin-dependent kinase inhibitor 1A (p21, Cip1)	Yes
	1312	COMT	catechol-O-methyltransferase	Yes
	1493	CTLA4	cytotoxic T-lymphocyte-associated protein 4	Yes
	1586	CYP17A1	cytochrome P450, family 17, subfamily A, polypeptide 1	Yes
	1543	CYP1A1	cytochrome P450, family 1, subfamily A, polypeptide 1	Yes
	1571	CYP2E1	cytochrome P450, family 2, subfamily E, polypeptide 1	Yes
	1906	EDN1	endothelin 1	Yes
	2052	EPHX1	epoxide hydrolase 1, microsomal (xenobiotic)	No
	2214	FCGR3A	Fc fragment of IgG, low affinity IIIa, receptor (CD16a)	Yes
	2944	GSTM1	glutathione S-transferase M1	Yes
	2950	GSTP1	glutathione S-transferase pi	Yes
	2952	GSTT1	glutathione S-transferase theta 1	No
	3105	HLA-A	major histocompatibility complex, class I, A	Yes
	3106	HLA-B	major histocompatibility complex, class I, B	Yes
	3107	HLA-C	major histocompatibility complex, class I, C	Yes
	3115	HLA-DPB1	major histocompatibility complex, class II, DP beta 1	Yes
	3117	HLA-DQA1	major histocompatibility complex, class II, DQ alpha 1	No
	3119	HLA-DQB1	major histocompatibility complex, class II, DQ beta 1	Yes
	3123	HLA-DRB1	major histocompatibility complex, class II, DR beta 1	Yes
	3553	IL1B	interleukin 1, beta	Yes
	3952	LEP	leptin	Yes
	3953	LEPR	leptin receptor	Yes
	4524	MTHFR	5,10-methylenetetrahydrofolate reductase (NADPH)	Yes
	4548	MTR	5-methyltetrahydrofolate-homocysteine methyltransferase	Yes
	4609	MYC	v-myc myelocytomatosis viral oncogene homolog (avian)	Yes
	9	NAT1	N-acetyltransferase 1 (arylamine N-acetyltransferase)	Yes
	10	NAT2	N-acetyltransferase 2 (arylamine N-acetyltransferase)	No
	1728	NQO1	NAD(P)H dehydrogenase, quinone 1	Yes
	5617	PRL	prolactin	Yes
	5981	RFC1	replication factor C (activator 1) 1, 145kDa	Yes
	6470	SHMT1	serine hydroxymethyltransferase 1 (soluble)	Yes
	7099	TLR4	toll-like receptor 4	Yes
	7124	TNF	tumor necrosis factor (TNF superfamily, member 2)	Yes
	7132	TNFRSF1A	tumor necrosis factor receptor superfamily, member 1A	Yes
	7133	TNFRSF1B	tumor necrosis factor receptor superfamily, member 1B	Yes
	7153	TOP2A	topoisomerase (DNA) II alpha 170kDa	Yes
	7157	TP53	tumor protein p53	Yes
	7298	TYMS	thymidylate synthetase	Yes
**Osteoarthritis**	9508	ADAMTS3	ADAM metallopeptidase with thrombospondin type 1 motif, 3	No
	176	ACAN	aggrecan	Yes
	367	AR	androgen receptor (dihydrotestosterone receptor; testicular feminization; spinal and bulbar muscular atrophy; Kennedy disease)	Yes
	54829	ASPN	asporin	No
	653	BMP5	bone morphogenetic protein 5	Yes
	801	CALM1	calmodulin 1 (phosphorylase kinase, delta)	Yes
	1301	COL11A1	collagen, type XI, alpha 1	Yes
	1302	COL11A2	collagen, type XI, alpha 2	Yes
	1277	COL1A1	collagen, type I, alpha 1	Yes
	1280	COL2A1	collagen, type II, alpha 1	Yes
	1297	COL9A1	collagen, type IX, alpha 1	Yes
	1298	COL9A2	collagen, type IX, alpha 2	Yes
	1299	COL9A3	collagen, type IX, alpha 3	Yes
	1311	COMP	cartilage oligomeric matrix protein	Yes
	5167	ENPP1	ectonucleotide pyrophosphatase/phosphodiesterase 1	Yes
	2099	ESR1	estrogen receptor 1	Yes

	2100	ESR2	estrogen receptor 2 (ER beta)	Yes
	2487	FRZB	frizzled-related protein	No
	1404	HAPLN1	hyaluronan and proteoglycan link protein 1	Yes
	3117	HLA-DQA1	major histocompatibility complex, class II, DQ alpha 1	No
	3119	HLA-DQB1	major histocompatibility complex, class II, DQ beta 1	Yes
	3123	HLA-DRB1	major histocompatibility complex, class II, DR beta 1	Yes
	3479	IGF1	insulin-like growth factor 1 (somatomedin C)	Yes
	3490	IGFBP7	insulin-like growth factor binding protein 7	Yes
	3552	IL1A	interleukin 1, alpha	Yes
	3553	IL1B	interleukin 1, beta	Yes
	3554	IL1R1	interleukin 1 receptor, type I	Yes
	3566	IL4R	interleukin 4 receptor	Yes
	3576	IL8	interleukin 8	Yes
	4041	LRP5	low density lipoprotein receptor-related protein 5	Yes
	4146	MATN1	matrilin 1, cartilage matrix protein	Yes
	4148	MATN3	matrilin 3	Yes
	4313	MMP2	matrix metallopeptidase 2 (gelatinase A, 72kDa gelatinase, 72kDa type IV collagenase)	Yes
	9060	PAPSS2	3'-phosphoadenosine 5'-phosphosulfate synthase 2	Yes
	12	SERPINA3	serpin peptidase inhibitor, clade A (alpha-1 antiproteinase, antitrypsin), member 3	Yes
	1836	SLC26A2	solute carrier family 26 (sulfate transporter), member 2	No
	7124	TNF	tumor necrosis factor (TNF superfamily, member 2)	Yes
	7130	TNFAIP6	tumor necrosis factor, alpha-induced protein 6	Yes
	7132	TNFRSF1A	tumor necrosis factor receptor superfamily, member 1A	Yes
	7133	TNFRSF1B	tumor necrosis factor receptor superfamily, member 1B	Yes
	7421	VDR	vitamin D (1,25- dihydroxyvitamin D3) receptor	Yes
**Myocardial ischemia**	19	ABCA1	ATP-binding cassette, sub-family A (ABC1), member 1	Yes
	1636	ACE	angiotensin I converting enzyme (peptidyl-dipeptidase A) 1	Yes
	177	AGER	advanced glycosylation end product-specific receptor	Yes
	183	AGT	angiotensinogen (serpin peptidase inhibitor, clade A, member 8)	Yes
	335	APOA1	apolipoprotein A-I	Yes
	116519	APOA5	apolipoprotein A-V	No
	338	APOB	apolipoprotein B (including Ag(x) antigen)	Yes
	348	APOE	apolipoprotein E	Yes
	1071	CETP	cholesteryl ester transfer protein, plasma	Yes
	2162	F13A1	coagulation factor XIII, A1 polypeptide	Yes
	2153	F5	coagulation factor V (proaccelerin, labile factor)	Yes
	2155	F7	coagulation factor VII (serum prothrombin conversion accelerator)	Yes
	2244	FGB	fibrinogen beta chain	Yes
	2811	GP1BA	glycoprotein Ib (platelet), alpha polypeptide	Yes
	3077	HFE	hemochromatosis	Yes
	3091	HIF1A	hypoxia-inducible factor 1, alpha subunit (basic helix-loop-helix transcription factor)	Yes
	3162	HMOX1	heme oxygenase (decycling) 1	Yes
	3673	ITGA2	integrin, alpha 2 (CD49B, alpha 2 subunit of VLA-2 receptor)	Yes
	3690	ITGB3	integrin, beta 3 (platelet glycoprotein IIIa, antigen CD61)	Yes
	3779	KCNMB1	potassium large conductance calcium-activated channel, subfamily M, beta member 1	Yes
	4018	LPA	lipoprotein, Lp(a)	Yes
	4023	LPL	lipoprotein lipase	Yes
	4314	MMP3	matrix metallopeptidase 3 (stromelysin 1, progelatinase)	Yes
	4524	MTHFR	5,10-methylenetetrahydrofolate reductase (NADPH)	Yes
	4846	NOS3	nitric oxide synthase 3 (endothelial cell)	Yes
	64805	P2RY12	purinergic receptor P2Y, G-protein coupled, 12	Yes
	5175	PECAM1	platelet/endothelial cell adhesion molecule (CD31 antigen)	Yes
	5444	PON1	paraoxonase 1	Yes
	5445	PON2	paraoxonase 2	Yes
	5446	PON3	paraoxonase 3	Yes
	5465	PPARA	peroxisome proliferator-activated receptor alpha	Yes
	5468	PPARG	peroxisome proliferator-activated receptor gamma	Yes
	5891	RAGE	renal tumor antigen	Yes
	6404	SELPLG	selectin P ligand	Yes
	5265	SERPINA1	serpin peptidase inhibitor, clade A (alpha-1 antiproteinase, antitrypsin), member 1	Yes
	5054	SERPINE1	serpin peptidase inhibitor, clade E (nexin, plasminogen activator inhibitor type 1), member 1	Yes
	7058	THBS2	thrombospondin 2	Yes
	7060	THBS4	thrombospondin 4	No

	57761	TRIB3	tribbles homolog 3 (Drosophila)	Yes
**Neural tube defects**	8854	ALDH1A2	aldehyde dehydrogenase 1 family, member A2	No
	328	APEX1	APEX nuclease (multifunctional DNA repair enzyme) 1	Yes
	635	BHMT	betaine-homocysteine methyltransferase	No
	23743	BHMT2	betaine-homocysteine methyltransferase 2	No
	652	BMP4	bone morphogenetic protein 4	Yes
	875	CBS	cystathionine-beta-synthase	Yes
	1381	CRABP1	cellular retinoic acid binding protein 1	No
	1382	CRABP2	cellular retinoic acid binding protein 2	Yes
	6372	CXCL6	chemokine (C-X-C motif) ligand 6 (granulocyte chemotactic protein 2)	Yes
	1592	CYP26A1	cytochrome P450, family 26, subfamily A, polypeptide 1	No
	56603	CYP26B1	cytochrome P450, family 26, subfamily B, polypeptide 1	No
	1806	DPYD	dihydropyrimidine dehydrogenase	No
	2068	ERCC2	excision repair cross-complementing rodent repair deficiency, complementation group 2 (xeroderma pigmentosum D)	Yes
	2346	FOLH1	folate hydrolase (prostate-specific membrane antigen) 1	No
	2350	FOLR2	folate receptor 2 (fetal)	No
	4397	MS	multiple sclerosis	No
	4522	MTHFD1	methylenetetrahydrofolate dehydrogenase (NADP+ dependent) 1, methenyltetrahydrofolate cyclohydrolase, formyltetrahydrofolate synthetase	Yes
	4524	MTHFR	5,10-methylenetetrahydrofolate reductase (NADPH)	Yes
	4548	MTR	5-methyltetrahydrofolate-homocysteine methyltransferase	Yes
	4552	MTRR	5-methyltetrahydrofolate-homocysteine methyltransferase reductase	No
	4594	MUT	methylmalonyl Coenzyme A mutase	No
	9241	NOG	noggin	Yes
	4968	OGG1	8-oxoguanine DNA glycosylase	Yes
	5110	PCMT1	protein-L-isoaspartate (D-aspartate) O-methyltransferase	Yes
	5156	PDGFRA	platelet-derived growth factor receptor, alpha polypeptide	Yes
	5981	RFC1	replication factor C (activator 1) 1, 145kDa	Yes
	6470	SHMT1	serine hydroxymethyltransferase 1 (soluble)	Yes
	6472	SHMT2	serine hydroxymethyltransferase 2 (mitochondrial)	Yes
	6947	TCN1	transcobalamin I (vitamin B12 binding protein, R binder family)	No
	6948	TCN2	transcobalamin II; macrocytic anemia	No
	7012	TERC	telomerase RNA component	No
	7298	TYMS	thymidylate synthetase	Yes
	7351	UCP2	uncoupling protein 2 (mitochondrial, proton carrier)	Yes
	7515	XRCC1	X-ray repair complementing defective repair in Chinese hamster cells 1	Yes
	7517	XRCC3	X-ray repair complementing defective repair in Chinese hamster cells 3	Yes
	7545	ZIC1	Zic family member 1 (odd-paired homolog, Drosophila)	Yes
	7546	ZIC2	Zic family member 2 (odd-paired homolog, Drosophila)	Yes
	7547	ZIC3	Zic family member 3 heterotaxy 1 (odd-paired homolog, Drosophila)	No
**Cervical carcinoma**	595	CCND1	cyclin D1	Yes
	1231	CCR2	chemokine (C-C motif) receptor 2	Yes
	1026	CDKN1A	cyclin-dependent kinase inhibitor 1A (p21, Cip1)	Yes
	1571	CYP2E1	cytochrome P450, family 2, subfamily E, polypeptide 1	Yes
	2052	EPHX1	epoxide hydrolase 1, microsomal (xenobiotic)	No
	355	FAS	Fas (TNF receptor superfamily, member 6)	Yes
	356	FASLG	Fas ligand (TNF superfamily, member 6)	Yes
	2272	FHIT	fragile histidine triad gene	Yes
	2944	GSTM1	glutathione S-transferase M1	Yes
	3105	HLA-A	major histocompatibility complex, class I, A	Yes
	3115	HLA-DPB1	major histocompatibility complex, class II, DP beta 1	Yes
	3117	HLA-DQA1	major histocompatibility complex, class II, DQ alpha 1	No
	3119	HLA-DQB1	major histocompatibility complex, class II, DQ beta 1	Yes
	3123	HLA-DRB1	major histocompatibility complex, class II, DR beta 1	Yes
	3265	HRAS	v-Ha-ras Harvey rat sarcoma viral oncogene homolog	Yes
	3451	IFNA17	interferon, alpha 17	No
	3458	IFNG	interferon, gamma	Yes
	3659	IRF1	interferon regulatory factor 1	Yes
	3802	KIR2DL1	killer cell immunoglobulin-like receptor, two domains, long cytoplasmic tail, 1	Yes
	3803	KIR2DL2	killer cell immunoglobulin-like receptor, two domains, long cytoplasmic tail, 2	Yes
	3804	KIR2DL3	killer cell immunoglobulin-like receptor, two domains, long cytoplasmic tail, 3	Yes
	3805	KIR2DL4	killer cell immunoglobulin-like receptor, two domains, long cytoplasmic tail, 4	Yes
	3809	KIR2DS4	killer cell immunoglobulin-like receptor, two domains, short cytoplasmic tail, 4	Yes

	3811	KIR3DL1	killer cell immunoglobulin-like receptor, three domains, long cytoplasmic tail, 1	Yes
	3812	KIR3DL2	killer cell immunoglobulin-like receptor, three domains, long cytoplasmic tail, 2	Yes
	115653	KIR3DL3	killer cell immunoglobulin-like receptor, three domains, long cytoplasmic tail, 3	No
	4276	MICA	MHC class I polypeptide-related sequence A	Yes
	4312	MMP1	matrix metallopeptidase 1 (interstitial collagenase)	Yes
	10	NAT2	N-acetyltransferase 2 (arylamine N-acetyltransferase)	No
	4968	OGG1	8-oxoguanine DNA glycosylase	Yes
	6556	SLC11A1	solute carrier family 11 (proton-coupled divalent metal ion transporters), member 1	No
	6768	ST14	suppression of tumorigenicity 14 (colon carcinoma)	Yes
	6890	TAP1	transporter 1, ATP-binding cassette, sub-family B (MDR/TAP)	Yes
	9338	TCEAL1	transcription elongation factor A (SII)-like 1	Yes
	7124	TNF	tumor necrosis factor (TNF superfamily, member 2)	Yes
	7157	TP53	tumor protein p53	Yes
	7161	TP73	tumor protein p73	Yes
	7515	XRCC1	X-ray repair complementing defective repair in Chinese hamster cells 1	Yes
**Epilepsy**	5243	ABCB1	ATP-binding cassette, sub-family B (MDR/TAP), member 1	No
	477	ATP1A2	ATPase, Na+/K+ transporting, alpha 2 (+) polypeptide	Yes
	627	BDNF	brain-derived neurotrophic factor	Yes
	773	CACNA1A	calcium channel, voltage-dependent, P/Q type, alpha 1A subunit	Yes
	8912	CACNA1H	calcium channel, voltage-dependent, T type, alpha 1H subunit	Yes
	1137	CHRNA4	cholinergic receptor, nicotinic, alpha 4	Yes
	1476	CSTB	cystatin B (stefin B)	Yes
	1557	CYP2C19	cytochrome P450, family 2, subfamily C, polypeptide 19	Yes
	1559	CYP2C9	cytochrome P450, family 2, subfamily C, polypeptide 9	Yes
	1621	DBH	dopamine beta-hydroxylase (dopamine beta-monooxygenase)	No
	11083	DIDO1	death inducer-obliterator 1	Yes
	2550	GABBR1	gamma-aminobutyric acid (GABA) B receptor, 1	Yes
	2558	GABRA5	gamma-aminobutyric acid (GABA) A receptor, alpha 5	No
	2560	GABRB1	gamma-aminobutyric acid (GABA) A receptor, beta 1	Yes
	2562	GABRB3	gamma-aminobutyric acid (GABA) A receptor, beta 3	Yes
	2563	GABRD	gamma-aminobutyric acid (GABA) A receptor, delta	Yes
	2566	GABRG2	gamma-aminobutyric acid (GABA) A receptor, gamma 2	Yes
	2897	GRIK1	glutamate receptor, ionotropic, kainate 1	Yes
	3123	HLA-DRB1	major histocompatibility complex, class II, DR beta 1	Yes
	3240	HP	haptoglobin	Yes
	3553	IL1B	interleukin 1, beta	Yes
	3766	KCNJ10	potassium inwardly-rectifying channel, subfamily J, member 10	Yes
	3760	KCNJ3	potassium inwardly-rectifying channel, subfamily J, member 3	Yes
	3763	KCNJ6	potassium inwardly-rectifying channel, subfamily J, member 6	Yes
	3765	KCNJ9	potassium inwardly-rectifying channel, subfamily J, member 9	Yes
	3785	KCNQ2	potassium voltage-gated channel, KQT-like subfamily, member 2	Yes
	3786	KCNQ3	potassium voltage-gated channel, KQT-like subfamily, member 3	Yes
	10656	KHDRBS3	KH domain containing, RNA binding, signal transduction associated 3	Yes
	163175	LGI4	leucine-rich repeat LGI family, member 4	No
	4128	MAOA	monoamine oxidase A	No
	4988	OPRM1	opioid receptor, mu 1	Yes
	5080	PAX6	paired box 6	Yes
	5173	PDYN	prodynorphin	No
	6323	SCN1A	sodium channel, voltage-gated, type I, alpha subunit	No
	6324	SCN1B	sodium channel, voltage-gated, type I, beta	Yes
	6508	SLC4A3	solute carrier family 4, anion exchanger, member 3	Yes
**Grave's disease**	720	C4A	complement component 4A (Rodgers blood group)	Yes
	958	CD40	CD40 molecule, TNF receptor superfamily member 5	Yes
	959	CD40LG	CD40 ligand (TNF superfamily, member 5, hyper-IgM syndrome)	Yes
	1493	CTLA4	cytotoxic T-lymphocyte-associated protein 4	Yes
	2099	ESR1	estrogen receptor 1	Yes
	2100	ESR2	estrogen receptor 2 (ER beta)	Yes
	356	FASLG	Fas ligand (TNF superfamily, member 6)	Yes
	2638	GC	group-specific component (vitamin D binding protein)	Yes
	3106	HLA-B	major histocompatibility complex, class I, B	Yes
	3117	HLA-DQA1	major histocompatibility complex, class II, DQ alpha 1	No
	3119	HLA-DQB1	major histocompatibility complex, class II, DQ beta 1	Yes
	3123	HLA-DRB1	major histocompatibility complex, class II, DR beta 1	Yes

	3383	ICAM1	intercellular adhesion molecule 1 (CD54), human rhinovirus receptor	Yes
	3458	IFNG	interferon, gamma	Yes
	3593	IL12B	interleukin 12B (natural killer cell stimulatory factor 2, cytotoxic lymphocyte maturation factor 2, p40)	Yes
	3596	IL13	interleukin 13	Yes
	3552	IL1A	interleukin 1, alpha	Yes
	3557	IL1RN	interleukin 1 receptor antagonist	Yes
	3565	IL4	interleukin 4	Yes
	3569	IL6	interleukin 6 (interferon, beta 2)	Yes
	3576	IL8	interleukin 8	Yes
	3630	INS	insulin	Yes
	3659	IRF1	interferon regulatory factor 1	Yes
	4049	LTA	lymphotoxin alpha (TNF superfamily, member 1)	Yes
	5698	PSMB9	proteasome (prosome, macropain) subunit, beta type, 9 (large multifunctional peptidase 2)	Yes
	26191	PTPN22	protein tyrosine phosphatase, non-receptor type 22 (lymphoid)	Yes
	117156	SCGB3A2	secretoglobin, family 3A, member 2	Yes
	5172	SLC26A4	solute carrier family 26, member 4	No
	387082	SUMO4	SMT3 suppressor of mif two 3 homolog 4 (S. cerevisiae)	Yes
	6890	TAP1	transporter 1, ATP-binding cassette, sub-family B (MDR/TAP)	Yes
	6891	TAP2	transporter 2, ATP-binding cassette, sub-family B (MDR/TAP)	Yes
	7038	TG	thyroglobulin	Yes
	7068	THRB	thyroid hormone receptor, beta (erythroblastic leukemia viral (v-erb-a) oncogene homolog 2, avian)	Yes
	7124	TNF	tumor necrosis factor (TNF superfamily, member 2)	Yes
	7253	TSHR	thyroid stimulating hormone receptor	Yes
	7421	VDR	vitamin D (1,25- dihydroxyvitamin D3) receptor	Yes
**Inflammatory bowel disease (IBD)**	5243	ABCB1	ATP-binding cassette, sub-family B (MDR/TAP), member 1	No
	1636	ACE	angiotensin I converting enzyme (peptidyl-dipeptidase A) 1	Yes
	324	APC	adenomatous polyposis coli	Yes
	335	APOA1	apolipoprotein A-I	Yes
	64127	NOD2	nucleotide-binding oligomerization domain containing 2	Yes
	929	CD14	CD14 molecule	Yes
	1493	CTLA4	cytotoxic T-lymphocyte-associated protein 4	Yes
	9231	DLG5	discs, large homolog 5 (Drosophila)	Yes
	2162	F13A1	coagulation factor XIII, A1 polypeptide	Yes
	2165	F13B	coagulation factor XIII, B polypeptide	Yes
	2147	F2	coagulation factor II (thrombin)	Yes
	3113	HLA-DPA1	major histocompatibility complex, class II, DP alpha 1	Yes
	3119	HLA-DQB1	major histocompatibility complex, class II, DQ beta 1	Yes
	3123	HLA-DRB1	major histocompatibility complex, class II, DR beta 1	Yes
	3383	ICAM1	intercellular adhesion molecule 1 (CD54), human rhinovirus receptor	Yes
	3458	IFNG	interferon, gamma	Yes
	3459	IFNGR1	interferon gamma receptor 1	Yes
	10261	IGSF6	immunoglobulin superfamily, member 6	No
	3586	IL10	interleukin 10	Yes
	3553	IL1B	interleukin 1, beta	Yes
	3554	IL1R1	interleukin 1 receptor, type I	Yes
	3557	IL1RN	interleukin 1 receptor antagonist	Yes
	3566	IL4R	interleukin 4 receptor	Yes
	3567	IL5	interleukin 5 (colony-stimulating factor, eosinophil)	Yes
	3683	ITGAL	integrin, alpha L (antigen CD11A (p180), lymphocyte function-associated antigen 1; alpha polypeptide)	Yes
	3695	ITGB7	integrin, beta 7	Yes
	3704	ITPA	inosine triphosphatase (nucleoside triphosphate pyrophosphatase)	No
	3998	LMAN1	lectin, mannose-binding, 1	Yes
	4292	MLH1	mutL homolog 1, colon cancer, nonpolyposis type 2 (E. coli)	Yes
	5327	PLAT	plasminogen activator, tissue	Yes
	5743	PTGS2	prostaglandin-endoperoxide synthase 2 (prostaglandin G/H synthase and cyclooxygenase)	Yes
	6556	SLC11A1	solute carrier family 11 (proton-coupled divalent metal ion transporters), member 1	No
	7040	TGFB1	transforming growth factor, beta 1	Yes
	7099	TLR4	toll-like receptor 4	Yes
	7124	TNF	tumor necrosis factor (TNF superfamily, member 2)	Yes
	7172	TPMT	thiopurine S-methyltransferase	No
**Atherosclerosis**	1636	ACE	angiotensin I converting enzyme (peptidyl-dipeptidase A) 1	Yes
	185	AGTR1	angiotensin II receptor, type 1	Yes

	240	ALOX5	arachidonate 5-lipoxygenase	Yes
	337	APOA4	apolipoprotein A-IV	Yes
	338	APOB	apolipoprotein B (including Ag(x) antigen)	Yes
	348	APOE	apolipoprotein E	Yes
	875	CBS	cystathionine-beta-synthase	Yes
	929	CD14	CD14 molecule	Yes
	1071	CETP	cholesteryl ester transfer protein, plasma	Yes
	1215	CMA1	chymase 1, mast cell	Yes
	1524	CX3CR1	chemokine (C-X3-C motif) receptor 1	Yes
	6387	CXCL12	chemokine (C-X-C motif) ligand 12 (stromal cell-derived factor 1)	Yes
	1535	CYBA	cytochrome b-245, alpha polypeptide	Yes
	1585	CYP11B2	cytochrome P450, family 11, subfamily B, polypeptide 2	Yes
	1571	CYP2E1	cytochrome P450, family 2, subfamily E, polypeptide 1	Yes
	2212	FCGR2A	Fc fragment of IgG, low affinity IIa, receptor (CD32)	Yes
	2944	GSTM1	glutathione S-transferase M1	Yes
	2952	GSTT1	glutathione S-transferase theta 1	No
	3077	HFE	hemochromatosis	Yes
	3569	IL6	interleukin 6 (interferon, beta 2)	Yes
	3630	INS	insulin	Yes
	4035	LRP1	low density lipoprotein-related protein 1 (alpha-2-macroglobulin receptor)	Yes
	4322	MMP13	matrix metallopeptidase 13 (collagenase 3)	Yes
	4314	MMP3	matrix metallopeptidase 3 (stromelysin 1, progelatinase)	Yes
	4353	MPO	myeloperoxidase	No
	4524	MTHFR	5,10-methylenetetrahydrofolate reductase (NADPH)	Yes
	4846	NOS3	nitric oxide synthase 3 (endothelial cell)	Yes
	5105	PCK1	phosphoenolpyruvate carboxykinase 1 (soluble)	No
	5444	PON1	paraoxonase 1	Yes
	5445	PON2	paraoxonase 2	Yes
	6401	SELE	selectin E (endothelial adhesion molecule 1)	Yes
	5054	SERPINE1	serpin peptidase inhibitor, clade E (nexin, plasminogen activator inhibitor type 1), member 1	Yes
	6720	SREBF1	sterol regulatory element binding transcription factor 1	Yes
	7099	TLR4	toll-like receptor 4	Yes
	7124	TNF	tumor necrosis factor (TNF superfamily, member 2)	Yes
**Ulcerative colitis**	5243	ABCB1	ATP-binding cassette, sub-family B (MDR/TAP), member 1	No
	7919	BAT1	HLA-B associated transcript 1	Yes
	7916	BAT2	HLA-B associated transcript 2	Yes
	64127	NOD2	nucleotide-binding oligomerization domain containing 2	Yes
	6356	CCL11	chemokine (C-C motif) ligand 11	Yes
	6369	CCL24	chemokine (C-C motif) ligand 24	Yes
	10344	CCL26	chemokine (C-C motif) ligand 26	Yes
	929	CD14	CD14 molecule	Yes
	1493	CTLA4	cytotoxic T-lymphocyte-associated protein 4	Yes
	3122	HLA-DRA	major histocompatibility complex, class II, DR alpha	Yes
	3123	HLA-DRB1	major histocompatibility complex, class II, DR beta 1	Yes
	3586	IL10	interleukin 10	Yes
	3589	IL11	interleukin 11	Yes
	3553	IL1B	interleukin 1, beta	Yes
	3554	IL1R1	interleukin 1 receptor, type I	Yes
	3557	IL1RN	interleukin 1 receptor antagonist	Yes
	3565	IL4	interleukin 4	Yes
	3845	KRAS	v-Ki-ras2 Kirsten rat sarcoma viral oncogene homolog	Yes
	4049	LTA	lymphotoxin alpha (TNF superfamily, member 1)	Yes
	4276	MICA	MHC class I polypeptide-related sequence A	Yes
	4282	MIF	macrophage migration inhibitory factor (glycosylation-inhibiting factor)	Yes
	4292	MLH1	mutL homolog 1, colon cancer, nonpolyposis type 2 (E. coli)	Yes
	4312	MMP1	matrix metallopeptidase 1 (interstitial collagenase)	Yes
	4314	MMP3	matrix metallopeptidase 3 (stromelysin 1, progelatinase)	Yes
	4584	MUC3A	mucin 3A, cell surface associated	No
	4790	NFKB1	nuclear factor of kappa light polypeptide gene enhancer in B-cells 1 (p105)	Yes
	4795	NFKBIL1	nuclear factor of kappa light polypeptide gene enhancer in B-cells inhibitor-like 1	Yes
	4842	NOS1	nitric oxide synthase 1 (neuronal)	Yes
	7941	PLA2G7	phospholipase A2, group VII (platelet-activating factor acetylhydrolase, plasma)	Yes
	7099	TLR4	toll-like receptor 4	Yes

	7124	TNF	tumor necrosis factor (TNF superfamily, member 2)	Yes
	7157	TP53	tumor protein p53	Yes
	7172	TPMT	thiopurine S-methyltransferase	No
	6955	TRA@	T cell receptor alpha locus	Yes
**Hypercholesterolaemia**	19	ABCA1	ATP-binding cassette, sub-family A (ABC1), member 1	Yes
	5243	ABCB1	ATP-binding cassette, sub-family B (MDR/TAP), member 1	No
	64240	ABCG5	ATP-binding cassette, sub-family G (WHITE), member 5 (sterolin 1)	Yes
	64241	ABCG8	ATP-binding cassette, sub-family G (WHITE), member 8 (sterolin 2)	Yes
	335	APOA1	apolipoprotein A-I	Yes
	337	APOA4	apolipoprotein A-IV	Yes
	116519	APOA5	apolipoprotein A-V	No
	338	APOB	apolipoprotein B (including Ag(x) antigen)	Yes
	345	APOC3	apolipoprotein C-III	No
	348	APOE	apolipoprotein E	Yes
	1071	CETP	cholesteryl ester transfer protein, plasma	Yes
	1535	CYBA	cytochrome b-245, alpha polypeptide	Yes
	1543	CYP1A1	cytochrome P450, family 1, subfamily A, polypeptide 1	Yes
	1557	CYP2C19	cytochrome P450, family 2, subfamily C, polypeptide 19	Yes
	1559	CYP2C9	cytochrome P450, family 2, subfamily C, polypeptide 9	Yes
	1565	CYP2D6	cytochrome P450, family 2, subfamily D, polypeptide 6	No
	1576	CYP3A4	cytochrome P450, family 3, subfamily A, polypeptide 4	No
	1577	CYP3A5	cytochrome P450, family 3, subfamily A, polypeptide 5	No
	1581	CYP7A1	cytochrome P450, family 7, subfamily A, polypeptide 1	No
	2169	FABP2	fatty acid binding protein 2, intestinal	No
	3700	ITIH4	inter-alpha (globulin) inhibitor H4 (plasma Kallikrein-sensitive glycoprotein)	No
	3949	LDLR	low density lipoprotein receptor (familial hypercholesterolemia)	Yes
	3990	LIPC	lipase, hepatic	Yes
	4023	LPL	lipoprotein lipase	Yes
	10	NAT2	N-acetyltransferase 2 (arylamine N-acetyltransferase)	No
	255738	PCSK9	proprotein convertase subtilisin/kexin type 9	No
	5444	PON1	paraoxonase 1	Yes
	5445	PON2	paraoxonase 2	Yes
	949	SCARB1	scavenger receptor class B, member 1	Yes
	6720	SREBF1	sterol regulatory element binding transcription factor 1	Yes
	6721	SREBF2	sterol regulatory element binding transcription factor 2	Yes
	7099	TLR4	toll-like receptor 4	Yes
**Endometrial carcinoma**	155	ADRB3	adrenergic, beta-3-, receptor	Yes
	367	AR	androgen receptor (dihydrotestosterone receptor; testicular feminization; spinal and bulbar muscular atrophy; Kennedy disease)	Yes
	673	BRAF	v-raf murine sarcoma viral oncogene homolog B1	Yes
	672	BRCA1	breast cancer 1, early onset	Yes
	595	CCND1	cyclin D1	Yes
	1312	COMT	catechol-O-methyltransferase	Yes
	1586	CYP17A1	cytochrome P450, family 17, subfamily A, polypeptide 1	Yes
	1588	CYP19A1	cytochrome P450, family 19, subfamily A, polypeptide 1	Yes
	1543	CYP1A1	cytochrome P450, family 1, subfamily A, polypeptide 1	Yes
	1544	CYP1A2	cytochrome P450, family 1, subfamily A, polypeptide 2	Yes
	1545	CYP1B1	cytochrome P450, family 1, subfamily B, polypeptide 1	Yes
	2067	ERCC1	excision repair cross-complementing rodent repair deficiency, complementation group 1 (includes overlapping antisense sequence)	Yes
	2068	ERCC2	excision repair cross-complementing rodent repair deficiency, complementation group 2 (xeroderma pigmentosum D)	Yes
	2072	ERCC4	excision repair cross-complementing rodent repair deficiency, complementation group 4	Yes
	2073	ERCC5	excision repair cross-complementing rodent repair deficiency, complementation group 5 (xeroderma pigmentosum, complementation group G (Cockayne syndrome))	Yes
	2099	ESR1	estrogen receptor 1	Yes
	2100	ESR2	estrogen receptor 2 (ER beta)	Yes
	3845	KRAS	v-Ki-ras2 Kirsten rat sarcoma viral oncogene homolog	Yes
	4292	MLH1	mutL homolog 1, colon cancer, nonpolyposis type 2 (E. coli)	Yes
	4436	MSH2	mutS homolog 2, colon cancer, nonpolyposis type 1 (E. coli)	Yes
	2956	MSH6	mutS homolog 6 (E. coli)	Yes
	4582	MUC1	mucin 1, cell surface associated	Yes

	5063	PAK3	p21 (CDKN1A)-activated kinase 3	Yes
	5241	PGR	progesterone receptor	Yes
	5728	PTEN	phosphatase and tensin homolog (mutated in multiple advanced cancers 1)	Yes
	7157	TP53	tumor protein p53	Yes
	7161	TP73	tumor protein p73	Yes
	54658	UGT1A1	UDP glucuronosyltransferase 1 family, polypeptide A1	No
	7507	XPA	xeroderma pigmentosum, complementation group A	Yes
	7508	XPC	xeroderma pigmentosum, complementation group C	Yes
	7515	XRCC1	X-ray repair complementing defective repair in Chinese hamster cells 1	Yes
	7517	XRCC3	X-ray repair complementing defective repair in Chinese hamster cells 3	Yes
	7520	XRCC5	X-ray repair complementing defective repair in Chinese hamster cells 5 (double-strand-break rejoining; Ku autoantigen, 80kDa)	Yes
**Migraine**	367	AR	androgen receptor (dihydrotestosterone receptor; testicular feminization; spinal and bulbar muscular atrophy; Kennedy disease)	Yes
	773	CACNA1A	calcium channel, voltage-dependent, P/Q type, alpha 1A subunit	Yes
	1493	CTLA4	cytotoxic T-lymphocyte-associated protein 4	Yes
	1621	DBH	dopamine beta-hydroxylase (dopamine beta-monooxygenase)	No
	11083	DIDO1	death inducer-obliterator 1	Yes
	1812	DRD1	dopamine receptor D1	Yes
	1814	DRD3	dopamine receptor D3	Yes
	1815	DRD4	dopamine receptor D4	Yes
	1816	DRD5	dopamine receptor D5	Yes
	1909	EDNRA	endothelin receptor type A	Yes
	1910	EDNRB	endothelin receptor type B	Yes
	114327	EFHC1	EF-hand domain (C-terminal) containing 1	Yes
	2099	ESR1	estrogen receptor 1	Yes
	2157	F8	coagulation factor VIII, procoagulant component (hemophilia A)	Yes
	3356	HTR2A	5-hydroxytryptamine (serotonin) receptor 2A	Yes
	3552	IL1A	interleukin 1, alpha	Yes
	3643	INSR	insulin receptor	Yes
	3782	KCNN3	potassium intermediate/small conductance calcium-activated channel, subfamily N, member 3	No
	192115	MA	Migraine with aura, susceptibility to	No
	4128	MAOA	monoamine oxidase A	No
	4129	MAOB	monoamine oxidase B	No
	4224	MEP1A	meprin A, alpha (PABA peptide hydrolase)	Yes
	4522	MTHFD1	methylenetetrahydrofolate dehydrogenase (NADP+ dependent) 1, methenyltetrahydrofolate cyclohydrolase, formyltetrahydrofolate synthetase	Yes
	4524	MTHFR	5,10-methylenetetrahydrofolate reductase (NADPH)	Yes
	4842	NOS1	nitric oxide synthase 1 (neuronal)	Yes
	4843	NOS2A	nitric oxide synthase 2A (inducible, hepatocytes)	Yes
	5241	PGR	progesterone receptor	Yes
	6005	RHAG	Rh-associated glycoprotein	Yes
	9481	SLC25A27	solute carrier family 25, member 27	No
	6532	SLC6A4	solute carrier family 6 (neurotransmitter transporter, serotonin), member 4	Yes
	7124	TNF	tumor necrosis factor (TNF superfamily, member 2)	Yes
	27242	TNFRSF21	tumor necrosis factor receptor superfamily, member 21	Yes
	7298	TYMS	thymidylate synthetase	Yes
**Pancreatitis**	125	ADH1B	alcohol dehydrogenase 1B (class I), beta polypeptide	No
	217	ALDH2	aldehyde dehydrogenase 2 family (mitochondrial)	Yes
	847	CAT	catalase	Yes
	929	CD14	CD14 molecule	Yes
	1080	CFTR	cystic fibrosis transmembrane conductance regulator (ATP-binding cassette sub-family C, member 7)	Yes
	2944	GSTM1	glutathione S-transferase M1	Yes
	2947	GSTM3	glutathione S-transferase M3 (brain)	Yes
	2950	GSTP1	glutathione S-transferase pi	Yes
	2952	GSTT1	glutathione S-transferase theta 1	No
	3105	HLA-A	major histocompatibility complex, class I, A	Yes
	3106	HLA-B	major histocompatibility complex, class I, B	Yes
	3107	HLA-C	major histocompatibility complex, class I, C	Yes
	3115	HLA-DPB1	major histocompatibility complex, class II, DP beta 1	Yes
	3119	HLA-DQB1	major histocompatibility complex, class II, DQ beta 1	Yes
	3123	HLA-DRB1	major histocompatibility complex, class II, DR beta 1	Yes
	3265	HRAS	v-Ha-ras Harvey rat sarcoma viral oncogene homolog	Yes

	3304	HSPA1B	heat shock 70kDa protein 1B	Yes
	3458	IFNG	interferon, gamma	Yes
	3586	IL10	interleukin 10	Yes
	3856	KRT8	keratin 8	Yes
	4257	MGST1	microsomal glutathione S-transferase 1	Yes
	5444	PON1	paraoxonase 1	Yes
	5644	PRSS1	protease, serine, 1 (trypsin 1)	Yes
	6648	SOD2	superoxide dismutase 2, mitochondrial	Yes
	6690	SPINK1	serine peptidase inhibitor, Kazal type 1	Yes
	7040	TGFB1	transforming growth factor, beta 1	Yes
	7124	TNF	tumor necrosis factor (TNF superfamily, member 2)	Yes
	54658	UGT1A1	UDP glucuronosyltransferase 1 family, polypeptide A1	No
	54578	UGT1A6	UDP glucuronosyltransferase 1 family, polypeptide A6	No
	54577	UGT1A7	UDP glucuronosyltransferase 1 family, polypeptide A7	No
	54576	UGT1A8	UDP glucuronosyltransferase 1 family, polypeptide A8	No
**Systemic scleroderma**	1636	ACE	angiotensin I converting enzyme (peptidyl-dipeptidase A) 1	Yes
	6347	CCL2	chemokine (C-C motif) ligand 2	Yes
	930	CD19	CD19 molecule	Yes
	1278	COL1A2	collagen, type I, alpha 2	Yes
	1281	COL3A1	collagen, type III, alpha 1 (Ehlers-Danlos syndrome type IV, autosomal dominant)	Yes
	1493	CTLA4	cytotoxic T-lymphocyte-associated protein 4	Yes
	1535	CYBA	cytochrome b-245, alpha polypeptide	Yes
	1557	CYP2C19	cytochrome P450, family 2, subfamily C, polypeptide 19	Yes
	1571	CYP2E1	cytochrome P450, family 2, subfamily E, polypeptide 1	Yes
	2200	FBN1	fibrillin 1	Yes
	2335	FN1	fibronectin 1	Yes
	3105	HLA-A	major histocompatibility complex, class I, A	Yes
	3115	HLA-DPB1	major histocompatibility complex, class II, DP beta 1	Yes
	3117	HLA-DQA1	major histocompatibility complex, class II, DQ alpha 1	No
	3119	HLA-DQB1	major histocompatibility complex, class II, DQ beta 1	Yes
	3123	HLA-DRB1	major histocompatibility complex, class II, DR beta 1	Yes
	3125	HLA-DRB3	major histocompatibility complex, class II, DR beta 3	Yes
	3126	HLA-DRB4	major histocompatibility complex, class II, DR beta 4	No
	3127	HLA-DRB5	major histocompatibility complex, class II, DR beta 5	Yes
	3552	IL1A	interleukin 1, alpha	Yes
	4312	MMP1	matrix metallopeptidase 1 (interstitial collagenase)	Yes
	4846	NOS3	nitric oxide synthase 3 (endothelial cell)	Yes
	5155	PDGFB	platelet-derived growth factor beta polypeptide (simian sarcoma viral (v-sis) oncogene homolog)	Yes
	5788	PTPRC	protein tyrosine phosphatase, receptor type, C	Yes
	6678	SPARC	secreted protein, acidic, cysteine-rich (osteonectin)	Yes
	6890	TAP1	transporter 1, ATP-binding cassette, sub-family B (MDR/TAP)	Yes
	6891	TAP2	transporter 2, ATP-binding cassette, sub-family B (MDR/TAP)	Yes
	7012	TERC	telomerase RNA component	No
	7040	TGFB1	transforming growth factor, beta 1	Yes
	7124	TNF	tumor necrosis factor (TNF superfamily, member 2)	Yes
	7133	TNFRSF1B	tumor necrosis factor receptor superfamily, member 1B	Yes
**Cirrhosis**	125	ADH1B	alcohol dehydrogenase 1B (class I), beta polypeptide	No
	126	ADH1C	alcohol dehydrogenase 1C (class I), gamma polypeptide	No
	217	ALDH2	aldehyde dehydrogenase 2 family (mitochondrial)	Yes
	718	C3	complement component 3	Yes
	1312	COMT	catechol-O-methyltransferase	Yes
	1586	CYP17A1	cytochrome P450, family 17, subfamily A, polypeptide 1	Yes
	1571	CYP2E1	cytochrome P450, family 2, subfamily E, polypeptide 1	Yes
	2052	EPHX1	epoxide hydrolase 1, microsomal (xenobiotic)	No
	2950	GSTP1	glutathione S-transferase pi	Yes
	3077	HFE	hemochromatosis	Yes
	3119	HLA-DQB1	major histocompatibility complex, class II, DQ beta 1	Yes
	3123	HLA-DRB1	major histocompatibility complex, class II, DR beta 1	Yes
	3162	HMOX1	heme oxygenase (decycling) 1	Yes
	3587	IL10RA	interleukin 10 receptor, alpha	Yes
	3552	IL1A	interleukin 1, alpha	Yes
	3553	IL1B	interleukin 1, beta	Yes

	3569	IL6	interleukin 6 (interferon, beta 2)	Yes
	3576	IL8	interleukin 8	Yes
	55605	KIF21A	kinesin family member 21A	No
	4049	LTA	lymphotoxin alpha (TNF superfamily, member 1)	Yes
	4353	MPO	myeloperoxidase	No
	4843	NOS2A	nitric oxide synthase 2A (inducible, hepatocytes)	Yes
	401	PHOX2A	paired-like homeobox 2a	Yes
	5265	SERPINA1	serpin peptidase inhibitor, clade A (alpha-1 antiproteinase, antitrypsin), member 1	Yes
	6648	SOD2	superoxide dismutase 2, mitochondrial	Yes
	6716	SRD5A2	steroid-5-alpha-reductase, alpha polypeptide 2 (3-oxo-5 alpha-steroid delta 4-dehydrogenase alpha 2)	No
	7037	TFRC	transferrin receptor (p90, CD71)	Yes
	7040	TGFB1	transforming growth factor, beta 1	Yes
	7124	TNF	tumor necrosis factor (TNF superfamily, member 2)	Yes
	7132	TNFRSF1A	tumor necrosis factor receptor superfamily, member 1A	Yes
