# Supplementary material for: Disease candidate gene identification and prioritization using protein interaction networks
Source: BMC Bioinformatics. 2009 Feb 27;10:73. doi: 10.1186/1471-2105-10-73 (PMC2657789; doi:10.1186/1471-2105-10-73)
Supplement: Additional file 3 — Cardiac septal defect associated OMIM records, genes and their immediate interactants (based on protein-protein interactions). [file 1471-2105-10-73-S3.pdf]

Additional File 3: This file has three tables. Table 1 has a list of 166 OMIM records associated with cardiac septal defects; Table 2 has a list of the corresponding 81 genes; Table 3 has a list of immediate interactants (based on human protein-protein interactions) of the 81 known cardiac septal defect associated genes.

Table 1: 166 OMIM records associated with cardiac septal defects. This list was generated by using the query "atrial septal defect" OR "ASD" OR "ventricular septal defect" OR "VSD" occurring either in the "Clinical Synopsis" or "allelic variants" sections of full OMIM records.

OMIM ID	OMIM Description
100300	Adams-Oliver Syndrome; Aos
101200	Apert Syndrome
106260	Ankyloblepharon-Ectodermal Defects-Cleft Lip/Palate
107480	Townes-Brocks Syndrome; Tbs
108800	Atrial Septal Defect 1; Asd1
108900	Atrial Septal Defect With Atrioventricular Conduction Defects
113000	Brachydactyly, Type B1; Bdb1
113301	Brachydactyly, Type E, With Atrial Septal Defect, Type Ii
117550	Sotos Syndrome
117650	Cerebrocostomandibular Syndrome
118450	Alagille Syndrome 1; Algs1
120160	Collagen, Type I, Alpha-2; Col1A2
121050	Contractural Arachnodactyly, Congenital
122850	Cranioacrofacial Syndrome
125520	Cayler Cardiofacial Syndrome
126320	Distichiasis With Congenital Anomalies Of The Heart And Peripheral Vasculature
134780	Femoral-Facial Syndrome; Ffs
135900	Coffin-Siris Syndrome
142900	Holt-Oram Syndrome; Hos
145410	Hypertelorism With Esophageal Abnormality And Hypospadias
146510	Pallister-Hall Syndrome; Phs
147791	Jacobsen Syndrome; Jbs
147920	Kabuki Syndrome
150250	Larsen Syndrome, Autosomal Dominant; Lrs1
156810	Microgastria-Limb Reduction Defects Association; Mlrd
160710	Myosin, Heavy Chain 6, Cardiac Muscle, Alpha; Myh6
162200	Neurofibromatosis, Type I; Nf1
163955	Noonan-Like/Multiple Giant Cell Lesion Syndrome
164210	Hemifacial Microsomia; Hfm
166500	Osteopathia Striata With Cranial Sclerosis; Oscs
169400	Pelger-Huet Anomaly; Pha

178370	Pulmonary Atresia With Ventricular Septal Defect
178650	Pulmonic Stenosis, Atrial Septal Defect, And Unique Electrocardiographic Abnormalities
182530	Son Of Sevenless, Drosophila, Homolog 1; Sos1
188400	Digeorge Syndrome; Dgs
190020	V-Ha-Ras Harvey Rat Sarcoma Viral Oncogene Homolog; Hras
190070	V-Ki-Ras2 Kirsten Rat Sarcoma Viral Oncogene Homolog; Kras
190198	Notch, Drosophila, Homolog Of, 1; Notch1
192430	Velocardiofacial Syndrome
194050	Williams-Beuren Syndrome; Wbs
194190	Wolf-Hirschhorn Syndrome; Whs
201000	Carpenter Syndrome
206900	Microphthalmia, Syndromic 3; Mcops3
207410	Antley-Bixler Syndrome; Abs
207620	Aphalangy With Hemivertebrae
208530	Asplenia With Cardiovascular Anomalies
209770	Aural Atresia, Multiple Congenital Anomalies, And Mental Retardation
210710	Microcephalic Osteodysplastic Primordial Dwarfism, Type I
211750	C Syndrome
212066	Congenital Disorder Of Glycosylation, Type Iia; Cdg2A
212090	Cardiac Septal Defects With Coarctation Of The Aorta
213980	Cerebrofaciothoracic Dysplasia
214800	Charge Syndrome
216340	Cleidocranial Dysplasia With Micrognathia, Absent Thumbs, And Distal Aphalangia
218040	Costello Syndrome
218649	Craniosynostosis-Mental Retardation Syndrome Of Lin And Gettig
220210	Dandy-Walker-Like Malformation With Atrioventricular Septal Defect
222448	Donnai-Barrow Syndrome
224700	Ebstein Anomaly
225500	Ellis-Van Creveld Syndrome; Evc
227255	Facial Dysmorphism With Multiple Malformations
228940	Fibuloulnar Aplasia Or Hypoplasia With Renal Abnormalities
229850	Fryns Syndrome; Frns
231060	Genitopalatocardiac Syndrome
235255	Mullerian Derivatives, Persistence Of, With Lymphangiectasia And Postaxial Polydactyly
235510	Hennekam Lymphangiectasia-Lymphedema Syndrome
235730	Mowat-Wilson Syndrome
235750	Hirschsprung Disease With Ulnar Polydactyly, Polysyndactyly Of Big Toes, And Ventricular Septal Defect
236680	Hydrolethalus Syndrome 1
241310	Hypomandibular Faciocranial Dysostosis

243800	Johanson-Blizzard Syndrome; Jbs
244300	Kapur-Toriello Syndrome
245150	Keutel Syndrome
245190	Kniest-Like Dysplasia, Lethal
248950	Mcdonough Syndrome
249630	Mental Retardation, Buenos Aires Type
249670	Mesoaxial Hexadactyly And Cardiac Malformation
250220	Spondylometaphyseal Dysplasia, Sedaghatian Type
256520	Neu-Laxova Syndrome; Nls
258315	Omodysplasia, Generalized Form
259770	Osteoporosis-Pseudoglioma Syndrome; Oppg
261540	Peters-Plus Syndrome
263630	Polysyndactyly With Cardiac Malformation
264480	Pseudotrisomy 13 Syndrome
268300	Roberts Syndrome; Rbs
269150	Schinzel-Giedion Midface-Retraction Syndrome
270100	Situs Inversus Viscerum
270400	Smith-Lemli-Opitz Syndrome; Slos
270460	Sonoda Syndrome
271640	Spondyloepimetaphyseal Dysplasia With Joint Laxity; Semdjl
272440	Syndactyly, Type I, With Microcephaly And Mental Retardation
274000	Thrombocytopenia-Absent Radius Syndrome
275210	Tight Skin Contracture Syndrome, Lethal
277740	White Forelock With Malformations
280000	Zunich Neuroectodermal Syndrome
300017	Filamin A; Flna
300166	Microphthalmia, Syndromic 2; Mcops2
300265	Zinc Finger Protein Of Cerebellum, 3; Zic3
300373	Osteopathia Striata With Cranial Sclerosis; Oscs
300463	Polyglutamine-Binding Protein 1; Pqbp1
300552	Midline 1; Mid1
301040	Alpha-Thalassemia/Mental Retardation Syndrome, Nondeletion Type, X-Linked; Atrx
302380	Catel-Manzke Syndrome
306955	Heterotaxy, Visceral, 1, X-Linked; Htx1
309500	Renpenning Syndrome 1; Rens1
309520	Lujan-Fryns Syndrome
309801	Microphthalmia, Syndromic 7; Mcops7
312870	Simpson-Golabi-Behmel Syndrome, Type 1; Sgbs1
314320	Trigonocephaly With Short Stature And Developmental Delay
600001	Pancreatic Hypoplasia, Congenital, With Diabetes Mellitus And Congenital Heart Disease
600024	Lamin B Receptor; Lbr

600123	Atrioventricular Septal Defect With Blepharophimosis And Anal And Radial Defects
600309	Atrioventricular Septal Defect; Avsd
600576	Gata-Binding Protein 4; Gata4
600584	Nk2 Homeobox 5; Nkx2-5
600987	Cardiac Malformation, Cleft Lip-Palate, Microcephaly And Digital Anomalies
601127	Fallot Complex With Severe Mental And Growth Retardation
601186	Microphthalmia, Syndromic 9; Mcops9
601284	Activin A Receptor, Type Ii-Like 1; Acvrl1
601321	Neurofibromatosis-Noonan Syndrome; Nfns
601322	Porencephaly, Cerebellar Hypoplasia, And Internal Malformations
601347	Myelodysplasia, Immunodeficiency, Facial Dysmorphism, Short Stature, And Psychomotor Delay
601348	Ectrodactyly Of Lower Limbs, Congenital Heart Defect, And Micrognathia
601355	Microcephaly, Congenital Heart Disease, Unilateral Renal Agenesis, And Hyposegmented Lungs
601357	Brachial Amelia, Forebrain Defects, And Facial Clefts
601450	Dislocation Of Hip, Congenital, With Hyperextensibility Of Fingers And Facial Dysmorphism
601601	Transcription Factor Ap2-Beta; Tfap2B
601620	T-Box 5; Tbx5
601803	Pallister-Killian Syndrome; Pks
601808	Chromosome 18Q Deletion Syndrome
601920	Jagged 1; Jag1
601927	Lymphedema, Cardiac Septal Defects, And Characteristic Facies
602054	T-Box 1; Tbx1
602337	Receptor Tyrosine Kinase-Like Orphan Receptor 2; Ror2
602535	Marshall-Smith Syndrome
602730	Activin A Receptor, Type Iib; Acvr2B
602937	Cbp/P300-Interacting Transactivator, With Glu/Asp-Rich C-Terminal Domain, 2; Cited2
603360	Peroxisome Biogenesis Factor 16; Pex16
605039	C-Like Syndrome
605321	Frontoocular Syndrome
605802	Zinc Finger E Box-Binding Homeobox 2; Zeb2
606061	T-Box 20; Tbx20
606170	Genitopatellar Syndrome
606519	Phace Association
607261	Evc2 Gene; Evc2
607323	Duane-Radial Ray Syndrome; Drrs
607343	Sal-Like 4; Sall4
607598	Lethal Congenital Contracture Syndrome 2; Lccs2

607872	Monosomy 1P36 Syndrome
608002	Nephrocystin 3; Nphp3
608227	Craniofacial Abnormalities, Cataracts, Congenital Heart Disease, Sacral Neural Tube Defects, And Growth And Developmental Retardation
608328	Weill-Marchesani Syndrome, Autosomal Dominant
608406	Vater-Like Defects With Pulmonary Hypertension, Laryngeal Webs, And Growth Deficiency
608454	Knobloch Syndrome, Type Ii
608571	Ulnar/Fibular Ray Defect And Brachydactyly
608572	Burn-Mckeown Syndrome
608688	Aicar Transformylase/Imp Cyclohydrolase, Deficiency Of
609029	Emanuel Syndrome
609192	Loeys-Dietz Syndrome, Type 1A; Lds1A
609625	Chromosome 10Q26 Deletion Syndrome
609654	Short Stature And Facioauriculothoracic Malformations
609945	Brachyphalangy, Polydactyly, And Tibial Aplasia/Hypoplasia
610338	Right Pulmonary Artery, Anomalous Origin Of, Familial
610745	Stimulated By Retinoic Acid 6, Mouse, Homolog Of; Stra6
611376	Mungan Syndrome; Mgs
611926	Immunodeficiency, Gonadal Dysgenesis, And Pulmonary Fibrosis

Table 2: Eighty-one human genes associated with 166 OMIM records (Table 1) that have a mention of cardiac septal defects ("atrial septal defect" OR “ASD” OR "ventricular septal defect" OR “VSD”) in the “Clinical Synopsis” or “allelic variants” sections of full OMIM records.

Gene ID	Gene Symbol	Gene Name
387569	ACF	Asymmetric crying facies (Cayler cardiofacial syndrome)
93	ACVR2B	activin A receptor, type IIB
94	ACVRL1	activin A receptor type II-like 1
100188340	AOS	Adams-Oliver syndrome
431	ASD1	atrial septal defect 1
471	ATIC	5-aminoimidazole-4-carboxamide ribonucleotide formyltransferase/IMP cyclohydrolase
546	ATRX	alpha thalassemia/mental retardation syndrome X-linked (RAD54 homolog, S. cerevisiae)
7446	AVSD1	atrioventricular septal defect 1
54880	BCOR	BCL6 co-repressor
10225	CD96	CD96 molecule
55636	CHD7	chromodomain helicase DNA binding protein 7
10370	CITED2	Cbp/p300-interacting transactivator, with Glu/Asp-rich carboxy-terminal domain, 2

1278	COL1A2	collagen, type I, alpha 2
1714	DGCR	DiGeorge syndrome chromosome region
1717	DHCR7	7-dehydrocholesterol reductase
8701	DNAH11	dynein, axonemal, heavy chain 11
2006	ELN	elastin
2065	ERBB3	v-erb-b2 erythroblastic leukemia viral oncogene homolog 3 (avian)
157570	ESCO2	establishment of cohesion 1 homolog 2 (S. cerevisiae)
2121	EVC	Ellis van Creveld syndrome
132884	EVC2	Ellis van Creveld syndrome 2
2200	FBN1	fibrillin 1
2201	FBN2	fibrillin 2
2263	FGFR2	fibroblast growth factor receptor 2
2316	FLNA	filamin A, alpha (actin binding protein 280)
2317	FLNB	filamin B, beta (actin binding protein 278)
2626	GATA4	GATA binding protein 4
2697	GJA1	gap junction protein, alpha 1, 43kDa
2737	GLI3	GLI-Kruppel family member GLI3
2719	GPC3	glypican 3
2969	GTF2I	general transcription factor II, i
9569	GTF2IRD1	GTF2I repeat domain containing 1
3052	HCCS	holocytochrome c synthase (cytochrome c heme-lyase)
170474	HFM	Hemifacial microsomia
3265	HRAS	v-Ha-ras Harvey rat sarcoma viral oncogene homolog
219844	HYLS1	hydrolethalus syndrome 1
8114	IV	inversus situs, viscerum
182	JAG1	jagged 1 (Alagille syndrome)
3719	JBS	Jacobsen syndrome
3826	KMS	Kabuki mental retardation syndrome
3845	KRAS	v-Ki-ras2 Kirsten rat sarcoma viral oncogene homolog
3930	LBR	lamin B receptor
4000	LMNA	lamin A/C
4036	LRP2	low density lipoprotein-related protein 2
4041	LRP5	low density lipoprotein receptor-related protein 5
4247	MGAT2	mannosyl (alpha-1,6-)-glycoprotein beta-1,2-N-acetylglucosaminyltransferase
4256	MGP	matrix Gla protein
100126595	MGS	Mungen syndrome
4281	MID1	midline 1 (Opitz/BBB syndrome)
4436	MSH2	mutS homolog 2, colon cancer, nonpolyposis type 1 (E. coli)
4624	MYH6	myosin, heavy chain 6, cardiac muscle, alpha
4763	NF1	neurofibromin 1
1482	NKX2-5	NK2 transcription factor related, locus 5 (Drosophila)

4851	NOTCH1	Notch homolog 1, translocation-associated (Drosophila)
27031	NPHP3	nephronophthisis 3 (adolescent)
64324	NSD1	nuclear receptor binding SET domain protein 1
8221	OGS2	Opitz G syndrome, type II
160176	OSCS	Osteopathia striata with cranial sclerosis
9409	PEX16	peroxisomal biogenesis factor 16
10084	PQBP1	polyglutamine binding protein 1
51715	RAB23	RAB23, member RAS oncogene family
4920	ROR2	receptor tyrosine kinase-like orphan receptor 2
6299	SALL1	sal-like 1 (Drosophila)
57167	SALL4	sal-like 4 (Drosophila)
9723	SEMA3E	sema domain, immunoglobulin domain (Ig), short basic domain, secreted, (semaphorin) 3E
6654	SOS1	son of sevenless homolog 1 (Drosophila)
6657	SOX2	SRY (sex determining region Y)-box 2
64220	STRA6	stimulated by retinoic acid gene 6 homolog (mouse)
100034701	TAR	Thrombocytopenia-absent radius syndrome
6899	TBX1	T-box 1
57057	TBX20	T-box 20
6910	TBX5	T-box 5
7021	TFAP2B	transcription factor AP-2 beta (activating enhancer binding protein 2 beta)
7046	TGFBR1	transforming growth factor, beta receptor 1
8626	TP63	tumor protein p63
780911	TQDS	Chromosome 10q deletion syndrome
197131	UBR1	ubiquitin protein ligase E3 component n-recognin 1
7467	WHCR	Wolf-Hirschhorn syndrome chromosome region
9839	ZEB2	zinc finger E-box binding homeobox 2
7547	ZIC3	Zic family member 3 (odd-paired homolog, Drosophila)
10269	ZMPSTE24	zinc metallopeptidase (STE24 homolog, S. cerevisiae)

Table 3: 479 genes (Test set) that are immediate interactants of the 81 human genes (Training set) associated with cardiac septal defects. Of these 48 genes are common between the training and test set. These are towards the end of the table and are highlighted in blue font.

**Entrez Gene ID**	**Gene Symbol**	**Gene Name**
10006	ABI1	abl-interactor 1
51205	ACP6	acid phosphatase 6, lysophosphatidic
91	ACVR1B	activin A receptor, type IB
186	AGTR2	angiotensin II receptor, type 2
26993	AKAP8L	A kinase (PRKA) anchor protein 8-like

213	ALB	albumin
239	ALOX12	arachidonate 12-lipoxygenase
242	ALOX12B	arachidonate 12-lipoxygenase, 12R type
57763	ANKRA2	ankyrin repeat, family A (RFXANK-like), 2
56899	ANKS1B	ankyrin repeat and sterile alpha motif domain containing 1B
163	AP2B1	adaptor-related protein complex 2, beta 1 subunit
320	APBA1	amyloid beta (A4) precursor protein-binding, family A, member 1
321	APBA2	amyloid beta (A4) precursor protein-binding, family A, member 2
322	APBB1	amyloid beta (A4) precursor protein-binding, family B, member 1 (Fe65)
54518	APBB1IP	amyloid beta (A4) precursor protein-binding, family B, member 1 interacting protein
338	APOB	apolipoprotein B (including Ag(x) antigen)
348	APOE	apolipoprotein E
350	APOH	apolipoprotein H (beta-2-glycoprotein I)
351	APP	amyloid beta (A4) precursor protein
367	AR	androgen receptor
369	ARAF	v-raf murine sarcoma 3611 viral oncogene homolog
9459	ARHGEF6	Rac/Cdc42 guanine nucleotide exchange factor (GEF) 6
445	ASS1	argininosuccinate synthetase 1
22926	ATF6	activating transcription factor 6
472	ATM	ataxia telangiectasia mutated
1822	ATN1	atrophin 1
529	ATP6V1E1	ATPase, H+ transporting, lysosomal 31kDa, V1 subunit E1
545	ATR	ataxia telangiectasia and Rad3 related
6310	ATXN1	ataxin 1
8312	AXIN1	axin 1
580	BARD1	BRCA1 associated RING domain 1
596	BCL2	B-cell CLL/lymphoma 2
604	BCL6	B-cell CLL/lymphoma 6
633	BGN	biglycan
641	BLM	Bloom syndrome
650	BMP2	bone morphogenetic protein 2
655	BMP7	bone morphogenetic protein 7
659	BMPR2	bone morphogenetic protein receptor, type II (serine/threonine kinase)
673	BRAF	v-raf murine sarcoma viral oncogene homolog B1
8315	BRAP	BRCA1 associated protein
672	BRCA1	breast cancer 1, early onset
675	BRCA2	breast cancer 2, early onset

695	BTK	Bruton agammaglobulinemia tyrosine kinase
776	CACNA 1D	calcium channel, voltage-dependent, L type, alpha 1D subunit
811	CALR	calreticulin
846	CASR	calcium-sensing receptor
857	CAV1	caveolin 1, caveolae protein, 22kDa
11335	CBX3	chromobox homolog 3 (HP1 gamma homolog, Drosophila)
23468	CBX5	chromobox homolog 5 (HP1 alpha homolog, Drosophila)
948	CD36	CD36 molecule (thrombospondin receptor)
960	CD44	CD44 molecule (Indian blood group)
3732	CD82	CD82 molecule
22918	CD93	CD93 molecule
993	CDC25 A	cell division cycle 25 homolog A (S. pombe)
998	CDC42	cell division cycle 42 (GTP binding protein, 25kDa)
1111	CHEK1	CHK1 checkpoint homolog (S. pombe)
11200	CHEK2	CHK2 checkpoint homolog (S. pombe)
10518	CIB2	calcium and integrin binding family member 2
163732	CITED4	Cbp/p300-interacting transactivator, with Glu/Asp-rich carboxy-terminal domain, 4
1191	CLU	clusterin
134147	CMBL	carboxymethylenebutenolidase homolog (Pseudomonas)
80790	CMIP	c-Maf-inducing protein
1272	CNTN1	contactin 1
50509	COL5A 3	collagen, type V, alpha 3
8904	CPNE1	copine I
221184	CPNE2	copine II
131034	CPNE4	copine IV
1387	CREBB P	CREB binding protein
1397	CRIP2	cysteine-rich protein 2
1398	CRK	v-crk sarcoma virus CT10 oncogene homolog (avian)
1399	CRKL	v-crk sarcoma virus CT10 oncogene homolog (avian)-like
1445	CSK	c-src tyrosine kinase
1453	CSNK1 D	casein kinase 1, delta
1499	CTNNB 1	catenin (cadherin-associated protein), beta 1, 88kDa
8029	CUBN	cubilin (intrinsic factor-cobalamin receptor)
1600	DAB1	disabled homolog 1 (Drosophila)
1601	DAB2	disabled homolog 2, mitogen-responsive phosphoprotein (Drosophila)
1634	DCN	decorin
8525	DGKZ	diacylglycerol kinase, zeta 104kDa

22943	DKK1	dickkopf homolog 1 (Xenopus laevis)
1739	DLG1	discs, large homolog 1 (Drosophila)
1740	DLG2	discs, large homolog 2, chapsyn-110 (Drosophila)
1741	DLG3	discs, large homolog 3 (neuroendocrine-dlg, Drosophila)
1742	DLG4	discs, large homolog 4 (Drosophila)
28514	DLL1	delta-like 1 (Drosophila)
54567	DLL4	delta-like 4 (Drosophila)
7266	DNAJC 7	DnaJ (Hsp40) homolog, subfamily C, member 7
1813	DRD2	dopamine receptor D2
1814	DRD3	dopamine receptor D3
113878	DTX2	deltex homolog 2 (Drosophila)
1869	E2F1	E2F transcription factor 1
1871	E2F3	E2F transcription factor 3
1874	E2F4	E2F transcription factor 4, p107/p130-binding
1950	EGF	epidermal growth factor (beta-urogastrone)
1956	EGFR	epidermal growth factor receptor (erythroblastic leukemia viral (v-erb-b) oncogene homolog, avian)
1974	EIF4A2	eukaryotic translation initiation factor 4A, isoform 2
1991	ELA2	elastase 2, neutrophil
1999	ELF3	E74-like factor 3 (ets domain transcription factor, epithelial-specific)
2010	EMD	emerin
2022	ENG	endoglin
2033	EP300	E1A binding protein p300
2038	EPB42	erythrocyte membrane protein band 4.2
54869	EPS8L1	EPS8-like 1
2064	ERBB2	v-erb-b2 erythroblastic leukemia viral oncogene homolog 2, neuro/glioblastoma derived oncogene homolog (avian)
55914	ERBB2I P	erbb2 interacting protein
2099	ESR1	estrogen receptor 1
9156	EXO1	exonuclease 1
54512	EXOSC 4	exosome component 4
2146	EZH2	enhancer of zeste homolog 2 (Drosophila)
2152	F3	coagulation factor III (thromboplastin, tissue factor)
3992	FADS1	fatty acid desaturase 1
355	FAS	Fas (TNF receptor superfamily, member 6)
54751	FBLIM1	filamin binding LIM protein 1
2192	FBLN1	fibulin 1
2199	FBLN2	fibulin 2
55294	FBXW7	F-box and WD repeat domain containing 7
2219	FCN1	ficolin (collagen/fibrinogen domain containing) 1
2246	FGF1	fibroblast growth factor 1 (acidic)

2255	FGF10	fibroblast growth factor 10
2247	FGF2	fibroblast growth factor 2 (basic)
2250	FGF5	fibroblast growth factor 5
2252	FGF7	fibroblast growth factor 7 (keratinocyte growth factor)
2254	FGF9	fibroblast growth factor 9 (glia-activating factor)
60681	FKBP10	FK506 binding protein 10, 65 kDa
2280	FKBP1 A	FK506 binding protein 1A, 12kDa
2335	FN1	fibronectin 1
23360	FNBP4	formin binding protein 4
2339	FNTA	farnesyltransferase, CAAX box, alpha
2353	FOS	v-fos FBJ murine osteosarcoma viral oncogene homolog
10818	FRS2	fibroblast growth factor receptor substrate 2
5045	FURIN	furin (paired basic amino acid cleaving enzyme)
2534	FYN	FYN oncogene related to SRC, FGR, YES
2549	GAB1	GRB2-associated binding protein 1
2638	GC	group-specific component (vitamin D binding protein)
10220	GDF11	growth differentiation factor 11
8200	GDF5	growth differentiation factor 5
10755	GIPC1	GIPC PDZ domain containing family, member 1
2700	GJA3	gap junction protein, alpha 3, 46kDa
2702	GJA5	gap junction protein, alpha 5, 40kDa
2811	GP1BA	glycoprotein Ib (platelet), alpha polypeptide
9737	GPRAS P1	G protein-coupled receptor associated sorting protein 1
29899	GPSM2	G-protein signaling modulator 2 (AGS3-like, C. elegans)
10750	GRAP	GRB2-related adaptor protein
2885	GRB2	growth factor receptor-bound protein 2
2886	GRB7	growth factor receptor-bound protein 7
2897	GRIK1	glutamate receptor, ionotropic, kainate 1
2899	GRIK3	glutamate receptor, ionotropic, kainate 3
2902	GRIN1	glutamate receptor, ionotropic, N-methyl D-aspartate 1
2914	GRM4	glutamate receptor, metabotropic 4
2915	GRM5	glutamate receptor, metabotropic 5
2917	GRM7	glutamate receptor, metabotropic 7
2918	GRM8	glutamate receptor, metabotropic 8
2932	GSK3B	glycogen synthase kinase 3 beta
9464	HAND2	heart and neural crest derivatives expressed 2
3065	HDAC1	histone deacetylase 1
3066	HDAC2	histone deacetylase 2
8841	HDAC3	histone deacetylase 3
9759	HDAC4	histone deacetylase 4
10014	HDAC5	histone deacetylase 5
10013	HDAC6	histone deacetylase 6

204851	HIPK1	homeodomain interacting protein kinase 1
28996	HIPK2	homeodomain interacting protein kinase 2
3182	HNRNP AB	heterogeneous nuclear ribonucleoprotein A/B
3183	HNRNP C	heterogeneous nuclear ribonucleoprotein C (C1/C2)
9987	HNRPD L	heterogeneous nuclear ribonucleoprotein D-like
3329	HSPD1	heat shock 60kDa protein 1 (chaperonin)
3476	IGBP1	immunoglobulin (CD79A) binding protein 1
3481	IGF2	insulin-like growth factor 2 (somatomedin A)
3547	IGSF1	immunoglobulin superfamily, member 1
9641	IKBKE	inhibitor of kappa light polypeptide gene enhancer in B-cells, kinase epsilon
22806	IKZF3	IKAROS family zinc finger 3 (Aiolos)
3572	IL6ST	interleukin 6 signal transducer (gp130, oncostatin M receptor)
3624	INHBA	inhibin, beta A
3625	INHBB	inhibin, beta B
3626	INHBC	inhibin, beta C
3630	INS	insulin
3643	INSR	insulin receptor
3654	IRAK1	interleukin-1 receptor-associated kinase 1
3656	IRAK2	interleukin-1 receptor-associated kinase 2
83737	ITCH	itchy E3 ubiquitin protein ligase homolog (mouse)
3673	ITGA2	integrin, alpha 2 (CD49B, alpha 2 subunit of VLA-2 receptor)
3674	ITGA2B	integrin, alpha 2b (platelet glycoprotein IIb of IIb/IIIa complex, antigen CD41)
3688	ITGB1	integrin, beta 1 (fibronectin receptor, beta polypeptide, antigen CD29 includes MDF2, MSK12)
9270	ITGB1B P1	integrin beta 1 binding protein 1
3695	ITGB7	integrin, beta 7
6453	ITSN1	intersectin 1 (SH3 domain protein)
3714	JAG2	jagged 2
3716	JAK1	Janus kinase 1 (a protein tyrosine kinase)
3720	JARID2	jumonji, AT rich interactive domain 2
3725	JUN	jun oncogene
8850	KAT2B	K(lysine) acetyltransferase 2B
338567	KCNK1 8	potassium channel, subfamily K, member 18
9816	KIAA01 33	KIAA0133
3932	LCK	lymphocyte-specific protein tyrosine kinase
26119	LDLRA P1	low density lipoprotein receptor adaptor protein 1

51176	LEF1	lymphoid enhancer-binding factor 1
3955	LFNG	LFNG O-fucosylpeptide 3-beta-N-acetylglucosaminyltransferase
3956	LGALS 1	lectin, galactoside-binding, soluble, 1
3958	LGALS 3	lectin, galactoside-binding, soluble, 3
9355	LHX2	LIM homeobox 2
8022	LHX3	LIM homeobox 3
4001	LMNB1	lamin B1
4015	LOX	lysyl oxidase
4018	LPA	lipoprotein, Lp(a)
4023	LPL	lipoprotein lipase
55805	LRP2BP	LRP2 binding protein
4043	LRPAP1	low density lipoprotein receptor-related protein associated protein 1
51691	LSM8	LSM8 homolog, U6 small nuclear RNA associated (S. cerevisiae)
4060	LUM	lumican
4069	LYZ	lysozyme (renal amyloidosis)
9500	MAGED 1	melanoma antigen family D, 1
9223	MAGI1	membrane associated guanylate kinase, WW and PDZ domain containing 1
9794	MAML1	mastermind-like 1 (Drosophila)
84441	MAML2	mastermind-like 2 (Drosophila)
55534	MAML3	mastermind-like 3 (Drosophila)
5604	MAP2K 1	mitogen-activated protein kinase kinase 1
6416	MAP2K 4	mitogen-activated protein kinase kinase 4
6885	MAP3K 7	mitogen-activated protein kinase kinase kinase 7
23118	MAP3K 7IP2	mitogen-activated protein kinase kinase kinase 7 interacting protein 2
1432	MAPK1 4	mitogen-activated protein kinase 14
5595	MAPK3	mitogen-activated protein kinase 3
5598	MAPK7	mitogen-activated protein kinase 7
5599	MAPK8	mitogen-activated protein kinase 8
9479	MAPK8 IP1	mitogen-activated protein kinase 8 interacting protein 1
23542	MAPK8 IP2	mitogen-activated protein kinase 8 interacting protein 2
4147	MATN2	matrilin 2
4149	MAX	MYC associated factor X
9656	MDC1	mediator of DNA damage checkpoint 1
4192	MDK	midkine (neurite growth-promoting factor 2)
4208	MEF2C	myocyte enhancer factor 2C

23184	MESDC 2	mesoderm development candidate 2
4237	MFAP2	microfibrillar-associated protein 2
8076	MFAP5	microfibrillar associated protein 5
4242	MFNG	MFNG O-fucosylpeptide 3-beta-N-acetylglucosaminyltransferase
11043	MID2	midline 2
4292	MLH1	mutL homolog 1, colon cancer, nonpolyposis type 2 (E. coli)
4300	MLLT3	myeloid/lymphoid or mixed-lineage leukemia (trithorax homolog, Drosophila); translocated to, 3
4301	MLLT4	myeloid/lymphoid or mixed-lineage leukemia (trithorax homolog, Drosophila); translocated to, 4
4318	MMP9	matrix metallopeptidase 9 (gelatinase B, 92kDa gelatinase, 92kDa type IV collagenase)
4361	MRE11 A	MRE11 meiotic recombination 11 homolog A (S. cerevisiae)
4437	MSH3	mutS homolog 3 (E. coli)
2956	MSH6	mutS homolog 6 (E. coli)
2660	MSTN	myostatin
4582	MUC1	mucin 1, cell surface associated
4609	MYC	v-myc myelocytomatosis viral oncogene homolog (avian)
58498	MYL7	myosin, light chain 7, regulatory
4653	MYOC	myocilin, trabecular meshwork inducible glucocorticoid response
9499	MYOT	myotilin
26502	NARF	nuclear prelamin A recognition factor
4683	NBN	nibrin
4690	NCK1	NCK adaptor protein 1
8440	NCK2	NCK adaptor protein 2
4692	NDN	necdin homolog (mouse)
51103	NDUFA F1	NADH dehydrogenase (ubiquinone) 1 alpha subcomplex, assembly factor 1
4776	NFATC 4	nuclear factor of activated T-cells, cytoplasmic, calcineurin-dependent 4
4790	NFKB1	nuclear factor of kappa light polypeptide gene enhancer in B-cells 1
4791	NFKB2	nuclear factor of kappa light polypeptide gene enhancer in B-cells 2 (p49/p100)
22795	NID2	nidogen 2 (osteonidogen)
4831	NME2	non-metastatic cells 2, protein (NM23B) expressed in
9722	NOS1A P	nitric oxide synthase 1 (neuronal) adaptor protein
4853	NOTCH 2	Notch homolog 2 (Drosophila)
4854	NOTCH 3	Notch homolog 3 (Drosophila)
4856	NOV	nephroblastoma overexpressed gene
4867	NPHP1	nephronophthisis 1 (juvenile)

7182	NR2C2	nuclear receptor subfamily 2, group C, member 2
2516	NR5A1	nuclear receptor subfamily 5, group A, member 1
3084	NRG1	neuregulin 1
9542	NRG2	neuregulin 2
8650	NUMB	numb homolog (Drosophila)
130497	OSR1	odd-skipped related 1 (Drosophila)
5034	P4HB	procollagen-proline, 2-oxoglutarate 4-dioxygenase (proline 4-hydroxylase), beta polypeptide
5036	PA2G4	proliferation-associated 2G4, 38kDa
29993	PACSIN 1	protein kinase C and casein kinase substrate in neurons 1
5080	PAX6	paired box 6
5111	PCNA	proliferating cell nuclear antigen
5147	PDE6D	phosphodiesterase 6D, cGMP-specific, rod, delta
5155	PDGFB	platelet-derived growth factor beta polypeptide (simian sarcoma viral (v-sis) oncogene homolog)
5824	PEX19	peroxisomal biogenesis factor 19
9063	PIAS2	protein inhibitor of activated STAT, 2
5290	PIK3CA	phosphoinositide-3-kinase, catalytic, alpha polypeptide
5294	PIK3CG	phosphoinositide-3-kinase, catalytic, gamma polypeptide
5295	PIK3R1	phosphoinositide-3-kinase, regulatory subunit 1 (alpha)
5296	PIK3R2	phosphoinositide-3-kinase, regulatory subunit 2 (beta)
23396	PIP5K1 C	phosphatidylinositol-4-phosphate 5-kinase, type I, gamma
5328	PLAU	plasminogen activator, urokinase
5335	PLCG1	phospholipase C, gamma 1
5371	PML	promyelocytic leukemia
5451	POU2F1	POU class 2 homeobox 1
5454	POU3F2	POU class 3 homeobox 2
5460	POU5F1	POU class 5 homeobox 1
5499	PPP1CA	protein phosphatase 1, catalytic subunit, alpha isoform
5520	PPP2R2 A	protein phosphatase 2 (formerly 2A), regulatory subunit B, alpha isoform
5521	PPP2R2 B	protein phosphatase 2 (formerly 2A), regulatory subunit B, beta isoform
5578	PRKCA	protein kinase C, alpha
5581	PRKCE	protein kinase C, epsilon
5592	PRKG1	protein kinase, cGMP-dependent, type I
5396	PRRX1	paired related homeobox 1
5657	PRTN3	proteinase 3
5663	PSEN1	presenilin 1
5664	PSEN2	presenilin 2 (Alzheimer disease 4)
9051	PSTPIP1	proline-serine-threonine phosphatase interacting protein 1
10728	PTGES3	prostaglandin E synthase 3 (cytosolic)

5747	PTK2	PTK2 protein tyrosine kinase 2
5753	PTK6	PTK6 protein tyrosine kinase 6
11156	PTP4A3	protein tyrosine phosphatase type IVA, member 3
10567	RABAC 1	Rab acceptor 1 (prenylated)
5879	RAC1	ras-related C3 botulinum toxin substrate 1 (rho family, small GTP binding protein Rac1)
10111	RAD50	RAD50 homolog (S. cerevisiae)
55698	RADIL	Rap GTPase interactor
5894	RAF1	v-raf-1 murine leukemia viral oncogene homolog 1
5898	RALA	v-ral simian leukemia viral oncogene homolog A (ras related)
5900	RALGD S	ral guanine nucleotide dissociation stimulator
5901	RAN	RAN, member RAS oncogene family
5910	RAP1G DS1	RAP1, GTP-GDP dissociation stimulator 1
5914	RARA	retinoic acid receptor, alpha
5921	RASA1	RAS p21 protein activator (GTPase activating protein) 1
5923	RASGR F1	Ras protein-specific guanine nucleotide-releasing factor 1
10235	RASGR P2	RAS guanyl releasing protein 2 (calcium and DAG-regulated)
115727	RASGR P4	RAS guanyl releasing protein 4
54922	RASIP1	Ras interacting protein 1
11186	RASSF1	Ras association (RalGDS/AF-6) domain family member 1
9770	RASSF2	Ras association (RalGDS/AF-6) domain family member 2
83593	RASSF5	Ras association (RalGDS/AF-6) domain family member 5
5925	RB1	retinoblastoma 1
5947	RBP1	retinol binding protein 1, cellular
3516	RBPJ	recombination signal binding protein for immunoglobulin kappa J region
5970	RELA	v-rel reticuloendotheliosis viral oncogene homolog A (avian)
5981	RFC1	replication factor C (activator 1) 1, 145kDa
266747	RGL4	ral guanine nucleotide dissociation stimulator-like 4
5999	RGS4	regulator of G-protein signaling 4
387	RHOA	ras homolog gene family, member A
9610	RIN1	Ras and Rab interactor 1
6014	RIT2	Ras-like without CAAX 2
6237	RRAS	related RAS viral (r-ras) oncogene homolog
6256	RXRA	retinoid X receptor, alpha
6271	S100A1	S100 calcium binding protein A1
7356	SCGB1 A1	secretoglobin, family 1A, member 1 (uteroglobin)
23513	SCRIB	scribbled homolog (Drosophila)

6401	SELE	selectin E
5054	SERPINE1	serpin peptidase inhibitor, clade E (nexin, plasminogen activator inhibitor type 1), member 1
10019	SH2B3	SH2B adaptor protein 3
30011	SH3KBP1	SH3-domain kinase binding protein 1
6464	SHC1	SHC (Src homology 2 domain containing) transforming protein 1
8036	SHOC2	soc-2 suppressor of clear homolog (C. elegans)
89790	SIGLEC10	sialic acid binding Ig-like lectin 10
6550	SLC9A3	solute carrier family 9 (sodium/hydrogen exchanger), member 3
4086	SMAD1	SMAD family member 1
4087	SMAD2	SMAD family member 2
4088	SMAD3	SMAD family member 3
4089	SMAD4	SMAD family member 4
4090	SMAD5	SMAD family member 5
4092	SMAD7	SMAD family member 7
8243	SMC1A	structural maintenance of chromosomes 1A
64750	SMURF2	SMAD specific E3 ubiquitin protein ligase 2
6626	SNRPA	small nuclear ribonucleoprotein polypeptide A
22938	SNW1	SNW domain containing 1
6642	SNX1	sorting nexin 1
112574	SNX18	sorting nexin 18
6643	SNX2	sorting nexin 2
58533	SNX6	sorting nexin 6
51429	SNX9	sorting nexin 9
6655	SOS2	son of sevenless homolog 2 (Drosophila)
6678	SPARC	secreted protein, acidic, cysteine-rich (osteonectin)
6690	SPINK1	serine peptidase inhibitor, Kazal type 1
6714	SRC	v-src sarcoma (Schmidt-Ruppin A-2) viral oncogene homolog (avian)
6720	SREBF1	sterol regulatory element binding transcription factor 1
6722	SRF	serum response factor (c-fos serum response element-binding transcription factor)
6732	SRPK1	SFRS protein kinase 1
6772	STAT1	signal transducer and activator of transcription 1, 91kDa
6774	STAT3	signal transducer and activator of transcription 3 (acute-phase response factor)
27148	STK36	serine/threonine kinase 36, fused homolog (Drosophila)
2040	STOM	stomatin
11171	STRAP	serine/threonine kinase receptor associated protein
51684	SUFU	suppressor of fused homolog (Drosophila)
7341	SUMO1	SMT3 suppressor of mif two 3 homolog 1 (S. cerevisiae)
23345	SYNE1	spectrin repeat containing, nuclear envelope 1

55333	SYNJ2BP	synaptojanin 2 binding protein
6860	SYT4	synaptotagmin IV
7013	TERF1	telomeric repeat binding factor (NIMA-interacting) 1
7020	TFAP2A	transcription factor AP-2 alpha (activating enhancer binding protein 2 alpha)
7022	TFAP2C	transcription factor AP-2 gamma (activating enhancer binding protein 2 gamma)
7038	TG	thyroglobulin
7040	TGFB1	transforming growth factor, beta 1
7042	TGFB2	transforming growth factor, beta 2
7043	TGFB3	transforming growth factor, beta 3
7045	TGFBI	transforming growth factor, beta-induced, 68kDa
7048	TGFBR2	transforming growth factor, beta receptor II (70/80kDa)
7049	TGFBR3	transforming growth factor, beta receptor III
9392	TGFBRAP1	transforming growth factor, beta receptor associated protein 1
7057	THBS1	thrombospondin 1
7067	THRA	thyroid hormone receptor, alpha (erythroblastic leukemia viral (v-erb-a) oncogene homolog, avian)
7074	TIAM1	T-cell lymphoma invasion and metastasis 1
7082	TJP1	tight junction protein 1 (zona occludens 1)
7094	TLN1	talin 1
7112	TMPO	thymopoietin
79155	TNIP2	TNFAIP3 interacting protein 2
1861	TOR1A	torsin family 1, member A (torsin A)
26092	TOR1AIP1	torsin A interacting protein 1
7186	TRAF2	TNF receptor-associated factor 2
11277	TREX1	three prime repair exonuclease 1
7204	TRIO	triple functional domain (PTPRF interacting)
7253	TSHR	thyroid stimulating hormone receptor
83942	TSSK1B	testis-specific serine kinase 1B
7265	TTC1	tetratricopeptide repeat domain 1
7276	TTR	transthyretin
7291	TWIST1	twist homolog 1 (Drosophila)
10907	TXNL4A	thioredoxin-like 4A
7329	UBE2I	ubiquitin-conjugating enzyme E2I (UBC9 homolog, yeast)
7391	USF1	upstream transcription factor 1
8409	UXT	ubiquitously-expressed transcript
7409	VAV1	vav 1 guanine nucleotide exchange factor

1462	VCAN	versican
7428	VHL	von Hippel-Lindau tumor suppressor
7450	VWF	von Willebrand factor
51729	WBP11	WW domain binding protein 11
55759	WDR12	WD repeat domain 12
55339	WDR33	WD repeat domain 33
51741	WWOX	WW domain containing oxidoreductase
331	XIAP	X-linked inhibitor of apoptosis
7532	YWHAG	tyrosine 3-monooxygenase/tryptophan 5-monooxygenase activation protein, gamma polypeptide
7528	YY1	YY1 transcription factor
51776	ZAK	sterile alpha motif and leucine zipper containing kinase AZK
7535	ZAP70	zeta-chain (TCR) associated protein kinase 70kDa
221527	ZBTB12	zinc finger and BTB domain containing 12
7709	ZBTB17	zinc finger and BTB domain containing 17
23414	ZFPM2	zinc finger protein, multitype 2
9372	ZFYVE9	zinc finger, FYVE domain containing 9
7545	ZIC1	Zic family member 1 (odd-paired homolog, Drosophila)
7546	ZIC2	Zic family member 2 (odd-paired homolog, Drosophila)
8187	ZNF239	zinc finger protein 239
84838	ZNF496	zinc finger protein 496
6657	SOX2	SRY (sex determining region Y)-box 2
7021	TFAP2B	transcription factor AP-2 beta (activating enhancer binding protein 2 beta)
3265	HRAS	v-Ha-ras Harvey rat sarcoma viral oncogene homolog
4041	LRP5	low density lipoprotein receptor-related protein 5
2006	ELN	elastin
54880	BCOR	BCL6 co-repressor
4851	NOTCH1	Notch homolog 1, translocation-associated (Drosophila)
2316	FLNA	filamin A, alpha (actin binding protein 280)
2317	FLNB	filamin B, beta (actin binding protein 278)
3845	KRAS	v-Ki-ras2 Kirsten rat sarcoma viral oncogene homolog
2626	GATA4	GATA binding protein 4
4000	LMNA	lamin A/C
10084	PQBP1	polyglutamine binding protein 1
2697	GJA1	gap junction protein, alpha 1, 43kDa
4256	MGP	matrix Gla protein
9409	PEX16	peroxisomal biogenesis factor 16
4920	ROR2	receptor tyrosine kinase-like orphan receptor 2
7046	TGFBR1	transforming growth factor, beta receptor 1
1482	NKX2-5	NK2 transcription factor related, locus 5 (Drosophila)
2201	FBN2	fibrillin 2

2200	FBN1	fibrillin 1
6299	SALL1	sal-like 1 (Drosophila)
2065	ERBB3	v-erb-b2 erythroblastic leukemia viral oncogene homolog 3 (avian)
94	ACVRL 1	activin A receptor type II-like 1
4763	NF1	neurofibromin 1
27031	NPHP3	nephronophthisis 3 (adolescent)
93	ACVR2 B	activin A receptor, type IIB
4036	LRP2	low density lipoprotein-related protein 2
2263	FGFR2	fibroblast growth factor receptor 2
4436	MSH2	mutS homolog 2, colon cancer, nonpolyposis type 1 (E. coli)
8626	TP63	tumor protein p63
9569	GTF2IR D1	GTF2I repeat domain containing 1
2737	GLI3	GLI-Kruppel family member GLI3
2719	GPC3	glypican 3
9839	ZEB2	zinc finger E-box binding homeobox 2
1717	DHCR7	7-dehydrocholesterol reductase
10370	CITED2	Cbp/p300-interacting transactivator, with Glu/Asp-rich carboxy-terminal domain, 2
182	JAG1	jagged 1 (Alagille syndrome)
4624	MYH6	myosin, heavy chain 6, cardiac muscle, alpha
546	ATRX	alpha thalassemia/mental retardation syndrome X-linked (RAD54 homolog, S. cerevisiae)
471	ATIC	5-aminoimidazole-4-carboxamide ribonucleotide formyltransferase/IMP cyclohydrolase
1278	COL1A 2	collagen, type I, alpha 2
3930	LBR	lamin B receptor
64324	NSD1	nuclear receptor binding SET domain protein 1
6910	TBX5	T-box 5
2969	GTF2I	general transcription factor II, i
6654	SOS1	son of sevenless homolog 1 (Drosophila)
4281	MID1	midline 1 (Opitz/BBB syndrome)
